# In which stage of the nursing process is ultrasonography used?

**DOI:** 10.1590/1980-220X-REEUSP-2025-0296en

**Published:** 2026-03-27

**Authors:** Vítor Monteiro Moraes, Taline Bavaresco, Miriam de Abreu Almeida, Daniele Delacanal Lazzari

**Affiliations:** 1 Universidade Federal de Santa Catarina, Programa de Pós-graduação em Enfermagem, Florianópolis, SC, Brazil.; 2 Universidade Federal do Rio Grande do Sul, Programa de Pós-graduação em Enfermagem, Porto Alegre, RS, Brazil.

**Keywords:** Nursing Process, Ultrasonography, Standardized Nursing Terminology, Models, Nursing, Nursing Care

## Abstract

**Objective:**

Reflecting on the inclusion of ultrasound in the steps of the Nursing Process.

**Method:**

This theoretical-reflective essay explores the relationship between ultrasound and the Nursing Process, based on the NANDA standardized nursing language systems NANDA *International, Nursing Interventions Classification* and *Nursing Outcomes Classification* and the five-step model of the Nursing Process (Assessment, Diagnosis, Planning, Implementation, and Evaluation).

**Results:**

A hypothetical clinical case is used as a guiding thread for reflection, presenting two lines of argument: the inclusion of ultrasound in the assessment stage (physical examination), or in the planning and implementation stages of the Nursing Process. The representational ambiguity of the five-stage theoretical model is demonstrated, and the possibilities and consequences of including ultrasound in one or another stage of the Nursing Process are discussed.

**Conclusion:**

Ultrasound challenges theoretical paradigms of the Nursing Process, and there is no definitive answer as to which stage it best fits into. Possible refinements to standardized nursing language systems are discussed, and further studies on the subject are suggested.

## INTRODUCTION

Ultrasonography (USG) performed by nurses was standardized by the Brazilian Federal Nursing Council (COFEN) in 2021^(1)^. It is recommended that this be carried out by properly trained nurses, prohibiting the issuance of reports or nosological diagnoses, at the bedside or in pre-hospital settings, aiming to guide procedures and identify phenomena that are sensitive to nursing. Therefore, the use of USG should occur within the context of the Nursing Process (NP)^(1,2)^.

In 2024, COFEN updated the resolution concerning the development of the NP, ratifying the five-step model: Assessment, Diagnosis, Planning, Implementation and Evaluation – ADPIE model^(2)^. Nursing diagnoses (ND), outcomes and indicators, interventions and activities can be supported by Standardized Nursing Language Systems (SNLS) and institutional protocols^(2)^. Among these, the NANDA International Nursing Diagnosis Classification (NANDA-I), *Nursing Interventions Classification* (NIC) and the *Nursing Outcomes Classification* (NOC)^(3,4,5)^ stand out.

However, the introduction of this technology into clinical practice challenges the conceptual boundaries of the NP itself. While USG provides objective data that qualifies the assessment stage, when used based on prior clinical judgment to guide conduct, it approaches a nursing intervention. This theoretical-practical ambiguity raises a necessary reflection: is bedside USG a means of assessment or does it, in itself, constitute a nursing intervention?

Reflecting on this issue is innovative for two main reasons. First, because it repositions USG within the theoretical field of nursing, testing the limits of established models, such as the ADPIE, and highlighting possible gaps in the representation of real practice. Secondly, because it prompts reinterpretation of standardized classifications (NANDA-I, NIC, NOC), suggesting that new categories or refinements may be necessary to encompass emerging practices.

Thus, this theoretical-reflective essay aims to reflect on the inclusion of USG in the stages of the NP in light of the SNLS and the ADPIE theoretical model. Through reflective analysis, this study seeks to unveil the implications of this insertion for the development of nursing science and to broaden the debate on technological innovation and the epistemology of care. It is understood that contemporary care practices, while they should be anchored in theoretical models that support, include, and explain them within the care process, frequently transcend the rigid limits of traditional theoretical frameworks.

## METHOD

This is a theoretical-reflective essay structured to answer the question: *Should USG performed by nurses within the NP be understood as a component of the assessment phase or as a nursing intervention?*

### Theoretical-Methodological Framework

The reflection was anchored in the organizational model of the NP (ADPIE) as a logical-procedural structure of care; in SNLS (NANDA-I, NIC, NOC), such as operational ontologies that link diagnosis-outcome-intervention; in the scientific representation/modeling literature, to discuss the limits and representational accuracy of models when confronted with practice; and contributions on clinical reasoning in nursing (analytical vs. non-analytical/pattern-based), to explain how bedside decisions can strain conceptual boundaries between assessing and intervening.

### Theoretical Corpus

The *corpus* brought together: (a) professional standards relevant to USG by nurses and the mandatory development of the NP; (b) current editions of the NANDA-I, NIC and NOC classifications; (c) publications on USG in nursing care/POCUS and on the development/validation of monitoring interventions; (d) reference works on scientific representation and clinical reasoning. The sufficiency of *corpus* was guided by theoretical saturation regarding the guiding question.

### Analytical Strategy

The analysis combined: (1) conceptual analysis (clarification of terms and relationships between NANDA-I/NIC/NOC categories in the context of ADPIE); (2) scenario contrast (USG as an extension of assessment versus USG as a nursing intervention, focusing on monitoring interventions); (3) a reflexive experiment with hypothetical clinical case in intensive care, used as a “test device” to test the explanatory performance of the ADPIE model under each framework; and (4) consequence analysis (implications of each framework for teaching, clinical practice and possible adjustments to the classifications).

### Construction and Use of the Hypothetical Case

The case was constructed from situations frequently encountered in hospital practice (post-operative care, risk of urinary retention, decision regarding bladder catheterization). Clinical data, eligible nursing diagnoses, expected outcomes, and possible interventions were explicitly stated. USG was incorporated as a decisive step to demonstrate, comparatively, how the framework, the assessment or the intervention alters the logical flow of the NP, the valuation of NOC indicators, and the selection of interventions in the NIC.

### Criteria of Rigor and Credibility

To ensure theoretical quality, the following were adopted: internal coherence of the argument; transparency in the chain of inferences; auditability of sources (direct citations to classifications and standards); conceptual triangulation between ADPIE, NANDA-I/NIC/NOC and scientific modeling literature; and deliberate search for counterexamples (e.g., situations where USG produces an “indirect effect” on outcomes).

### Ethical Aspects

As it was a theoretical-reflexive study, the research was not submitted for consideration to a Research Ethics Committee. The clinical case used for illustration is hypothetical and does not refer to any real patient. Furthermore, good academic practices for knowledge production are strictly adhered to, ensuring correct citation and proper referencing of all sources used.

## IS ULTRASONOGRAPHY PART OF THE NURSING PHYSICAL EXAMINATION, BEING PART OF THE BEDSIDE PATIENT ASSESSMENT STAGE?

USG allows the visualization and interpretation of the human body structures, characterizing them and allowing inferences about their function. This, in itself, is characterized as an extension of the physical examination^(6)^. Nursing assessment can be performed using instruments, including imaging tests^(2)^. NANDA-I systematically and structurally describes the entire reasoning process from assessment to identification of the priority nursing diagnosis, and USG can be included in screening and in-depth evaluations^(3)^.

The screening assessment is considered the initial phase of the evaluation, in which the first data are collected and prompt the start of clinical reasoning. Therefore, USG can enrich this moment as a tool that supports and enhances clinical reasoning^(3)^. The evaluation process is directed and deepened based on the screening data, resulting in a ND^(3)^. If USG is performed in response to a need noticed in screening, it can provide objective information that guides specific diagnostic tests. This also makes it possible to see that NANDA-I advocates hypothetical-deductive thinking, by providing for the use of specific data collection procedures to confirm or refute diagnostic hypotheses^(3)^.

In addition to the arguments already presented, it is important to understand how USG fits into the ADPIE model within this line of reasoning. In a hypothetical scenario, a nurse arrives at the Intensive Care Unit (ICU) at 1 p.m. for a six-hour shift. He/she receives the case of an 80-year-old male patient who underwent abdominal surgery, is bedridden, and has benign prostatic hyperplasia (BPH) as a comorbidity. During the patient assessment, the nurse notices that the indwelling urinary catheter (IUC) has been removed, finding, in the nursing records, that this occurred at 12:00 (letter A of the ADPIE model). The nursing diagnosis defined was “Risk of Urinary Retention (00322)”, whose risk factors in this case are “inadequate posture on the toilet” and “inadequate privacy”, and as an associated condition, “prostate diseases”. This comprises the diagnostic stage, letter D^(3)^.

In the planning stage (letter P), the nurse lists the NOC outcome “Urinary Elimination (0503)” and the NIC intervention “Assistance with self-care: use of the toilet (1804)”^(4,5)^. This intervention includes assistance with urinary elimination, provision of assistive devices when necessary, and assurance of privacy. The clinical indicators, valued on a 5-point Likert scale, where the higher the score, the more desirable the state, are described in Chart 1 ^(5)^.

**Chart 1:** Indicators of nursing outcome “Urinary Elimination (0503)” chosen for the hypothetical case – Florianópolis, SC, Brazil, 2025.

Indicator	Measurement scale				
	1. Seriously compromised	2. Very compromised	3. Moderately compromised	4. Slightly compromised	5. Not compromised
Recognition of urge					X O
Elimination pattern					X O
Amount of urine					X O
Empties bladder completely					X O
	1 Severe	2 Substantial	3 Moderate	4 Mild	5 None
Urinary retention					X O

Note: “X” represents the patient’s current state and “O” the expected state. Source: Adapted from Moorhead, Johnson and Swanson (2024, pp. 408–409)^(5)^.

In this hypothetical case, the implementation of the care plan throughout the nurse’s shift is considered complete, since the care activities were carried out without any noteworthy incidents. Thus, stage I is complete.

Nursing evolution, the next and final stage of the NP, is described as the “evaluation of the nursing and health outcomes achieved for the individual, family, community, and special groups”^(2)^, and considers that this step “allows for the analysis and review of the entire Nursing Process”^(2)^. It is during evolution phase that nursing outcome indicators are evaluated and their behavior is analyzed based on the care plan implemented^(2,5)^ (Chart 2).

**Chart 2:** Scores achieved in the nursing outcome indicators “Urinary Elimination (0503)” valued at 6:00 PM – Florianópolis, SC, Brazil, 2025.

Indicator	Measurement scale					
	1. Seriously compromised	2. Very compromised	3. Moderately compromised	4. Slightly compromised	5. Not compromised	NA*
Recognition of urge	X					
Elimination pattern	X					
Amount of urine						X
Empties bladder completely	X					
	1 Severe	2 Substantial	3 Moderate	4 Mild	5 None	
Urinary retention	X					

Note: NA*: Not assessed. “X” represents the score achieved. Source: Adapted from Moorhead, Johnson and Swanson (2024, pp. 408–409)^(5)^.

At the 6 pm assessment, the patient scored 1 in “recognition of urge” due to the absence of urination and in “elimination pattern” because they had not urinated in six hours^(5)^. The “amount of urine” was not assessed because the patient did not urinate, and evaluation of the suprapubic region by palpation and percussion is hampered by the abdominal surgical wound. The fact that the patient did not empty their bladder and, when offered a urinal, reported not being able to urinate, justifies the score of 1 in the indicators “empties bladder completely” and “urinary retention”^(5)^.

The nurse then decides to assess the patient with an USG. When this happens, the NP restarts, resuming the first stage of ADPIE. An USG of the bladder reveals a significant amount of urine retained in the bladder. This allows us to identify how USG relates to the “in-depth assessment” stage according to NANDA-I, by supporting clinical reasoning and diagnosis^(3)^.

Thus, the ND changes to “Impaired urinary elimination (00016)”, which is defined as “inability to effectively excrete fluids and waste stored in the bladder through the urethra”^(3)^. The defining characteristic found is “urinary retention,” with related factors being “improper posture on the toilet” and “inadequate privacy”^(3)^. The patient is still in a high-risk population due to being older, and has associated conditions such as BPH^(3)^.

In the planning phase, the nurse revisits the clinical picture to identify the patient’s current state and list interventions that will allow them to achieve the expected results^(2)^. The patient’s current condition is the same as that presented in Chart 2, plus the findings provided by the ultrasonography (Chart 3).

**Chart 3:** Planning stage after bladder USG assessment – Florianópolis, SC, Brazil, 2025.

Indicator	Measurement scale				
	1. Seriously compromised	2. Very compromised	3. Moderately compromised	4. Slightly compromised	5. Not compromised
Recognition of urge	X				O
Elimination pattern	X				O
Amount of urine					X O
Empties bladder completely	X				O
	1 Severe	2 Substantial	3 Moderate	4 Mild	5 None
Urinary retention	X				O

Note: “X” represents the patient’s current state and “O” the expected state. Source: Adapted from Moorhead, Johnson and Swanson (2024, pp. 408–409)^(5)^.

There was a change in the indicators evaluated after applying USG in this case. The indicator “urine amount”, which had not been evaluated, now shows a score of 5, and this occurred because the USG allowed for the non-invasive measurement of bladder volume. Furthermore, it allowed for a more consistent assessment of the indicators “empties bladder completely” and “urinary retention”^(5)^.

Therefore, the nurse decides to perform the intervention “Bladder Catheterization (0580)”, directing the intervention to the defining characteristic “urinary retention” and no longer to the related factors^(3,4)^. The volume of urine drained was 550 milliliters. From this, it is possible to reassess the scores achieved in each clinical indicator of the outcome “Urinary elimination (0503)”^(5)^ (Chart 4).

**Chart 4:** Scores obtained in the clinical indicators evaluated in the case after bladder catheterization – Florianópolis, SC, Brazil, 2025.

Indicator	Measurement scale				
	1. Seriously compromised	2. Very compromised	3. Moderately compromised	4. Slightly compromised	5. Not compromised
Recognition of urge	X				
Elimination pattern			X		
Amount of urine					X
Empties bladder completely					X
	1 Severe	2 Substantial	3 Moderate	4 Mild	5 None
Urinary retention					X

Note: “X” represents the score achieved. Source: Adapted from Moorhead, Johnson and Swanson (2024, pp. 408–409)^(5)^.

At this point, again in step E of the ADPIE model, the results obtained are compared. The indicator “recognition of urge” remains at a score of 1, as the patient presented with retained urine in the bladder and did not report the urge to urinate or discomfort. The “elimination pattern” is partially restored from catheterization, assessed as moderately compromised (score 3). The “urine amount” indicator was not modified, as the USG showed an adequate amount of urine, which was confirmed by catheterization. The indicators “empties bladder completely” and “urinary retention” are fully resolved as a result of the intervention performed. This concludes the description of the mental exercise with the application of USG in the ADPIE model in the first stage of the NP, the assessment^(5)^.

This case demonstrates that USG fits into the evaluation stage. Furthermore, USG does not meet the NIC definition of nursing intervention, which is “any treatment that, based on clinical judgment and knowledge, a nurse puts into practice to improve patient outcomes”^(4)^. In this sense, USG is not a “treatment” because it did not directly modify nursing outcomes.

## IS ULTRASONOGRAPHY A NURSING INTERVENTION?

Since 1996, the NIC has considered USG performed by nurses to be a nursing intervention. “Ultrasonography: Obstetric and Gynecological (6982)”^(4)^. Currently in its eighth edition, there is a second nursing intervention using USG: “Ultrasonography: bladder (0565)”^(4,5,6,7)^. This last one was developed recently^(7)^ and is defined as “performing ultrasonography examinations to determine the function or structure of the bladder”^(4)^.

Adding to the NIC definition of nursing intervention, NANDA-I, in describing the Tripartite Model by Kamitsuru, states that “autonomous nursing actions or interventions are *treatments* based on nursing knowledge standards, determined by the nurse as appropriate to address the etiological factors of a nursing diagnosis or manage symptoms^(3)^ (emphasis added). Following this line of reasoning, USG can be considered a treatment, as it is directed at the ND and improves outcomes.

The term “treatment” is not formally described in NIC or the NANDA-I classification. In the Online Portuguese Dictionary, one of the meanings of the entry “treatment” is “the set of practical means to combat a disease; therapy”^(8)^. The Michaelis Dictionary, also online, lists one of the meanings of this same entry as “the set of means used by a doctor to cure or relieve the patient”^(9)^.

In common, the linguistic meanings of the term “treatment” include the “set of means”. In this sense, USG performed by nurses is part of the “set of means,” and is, in itself, also a “set of means” or actions that allow for better results, this being its “therapeutic effect,” which denotes the idea of “treatment.” It is also important to point out that this “effect,” in this case, is not direct, but rather indirect. This is because interventions using USG can be considered monitoring interventions, as described in NIC^(4)^.

NIC includes numerous monitoring interventions, such as acid-base and vital sign monitoring, which incorporate and reflect clinical judgment and indicate to the nurse what to look for and what to do when an event occurs^(4)^.

Furthermore, it is important to highlight that risk diagnoses require monitoring interventions. NANDA-I defines risky ND as the susceptibility to develop an undesirable human response in the future^(3)^. In addition to acting to control risk factors, it is also necessary to identify the occurrence of the event at risk. In a quick example, the diagnosis “Risk of pressure injury in adults (00304)” can be directly modified or mitigated by the intervention “Pressure injury prevention (3540)” through the control of risk factors, and the occurrence of the problem-focused diagnosis – “Pressure injury in adults (00312)” – can be verified by the monitoring intervention “Skin supervision (3590)”^(3,4)^.

For example, to carry out the intervention “Vital signs monitoring (6680)”, it is necessary to master techniques such as measuring blood pressure, peripheral oxygen saturation, body temperature, heart rate, etc.^(4)^. This “know-how” is what defines the scope of the intervention. This is an intervention preceded by clinical reasoning that underpins and prompts its implementation in the care setting to achieve specific results^(4)^.

In contrast to the previous section, a mental exercise of fitting USG into the ADPIE model is performed, this time as a nursing intervention, using the same hypothetical case. Subsequently, with the nursing diagnosis “Risk of Urinary Retention (00322)”, NOC “Urinary Elimination (0503)” and NIC “Assistance with self-care: use of the toilet (1804)”^(3,4,5)^, at 6:00 pm on the day in question, the patient presents the evaluated scores already described in Chart 2.

When evaluating these scores, the nurse finds that the patient’s ND remains the same, “Risk of Urinary Retention (00322)”^(3)^. This is because it is possible to judge that there is not sufficient evidence to warrant a change, since no new risk factors have emerged and the existing ones have not been resolved. Furthermore, the occurrence of urinary retention can be better assessed using the nursing intervention “Ultrasonography: bladder (0565)”, since just as the patient may be presenting with “Impaired Urinary Elimination (00016)”, it is also possible that there has been a reduction in urine production^(3,4)^. The difference is that, in one case, performing a bladder catheterization is an intervention that can be considered; in the other, it cannot.

Thus, when achieving the result “Urinary Elimination (0503)”, the nurse lists the nursing intervention “Ultrasonography: bladder (0565)”. At this planning stage, the intervention “Bladder catheterization (0580)” could also be listed, to be performed depending on the result achieved by the intervention “Ultrasonography: bladder (0565)”^(4,5)^. Therefore, in this exercise, at this time, only the intervention “Ultrasonography: bladder (0565)”^(4)^ (Chart 5) is proposed.

**Chart 5:** Current state of the patient before the nursing intervention “Ultrasonography: bladder (0565)” and the expected state – Florianópolis, SC, Brazil, 2025.

Indicator	Measurement scale					
	1. Seriously compromised	2. Very compromised	3. Moderately compromised	4. Slightly compromised	5. Not compromised	NA*
Recognition of urge	X				O	
Elimination pattern	X				O	
Amount of urine					O	X
Empties bladder completely	X				O	
	1 Severe	2 Substantial	3 Moderate	4 Mild	5 None	NA*
Urinary retention	X				O	

Note: NA*: Not assessed. “X” represents the patient’s current state and “O” the expected state. Source: Adapted from Moorhead, Johnson and Swanson (2024, pp. 408–409)^(5)^.

Unlike the previous line of reasoning, it is clear that the current state is the same as the stage of evolution in the cycle that initiated this exercise. The implementation phase occurs through the intervention “Ultrasonography: bladder (0565)”^(4)^. During the intervention, the nurse identified an increased urine volume based on the USG. Thus, the nursing outcome can be assessed again (Chart 6).

**Chart 6:** Scores achieved by the patient after the nursing intervention “Ultrasonography: bladder (0565)” – Florianópolis, SC, Brazil, 2025.

Indicator	Measurement scale					
	1. Seriously compromised	2. Very compromised	3. Moderately compromised	4. Slightly compromised	5. Not compromised	NA*
Recognition of urge	X					
Elimination pattern	X					
Amount of urine					X	
Empties bladder completely	X					
	1 Severe	2 Substantial	3 Moderate	4 Mild	5 None	NA*
Urinary retention	X					

Note: NA*: Not assessed. “X” represents the score achieved. Source: Adapted from Moorhead, Johnson and Swanson (2024, pp. 408–409)^(5)^.

Based on the scores obtained, it is possible to identify that the “amount of urine” indicator after performing the USG can be evaluated and is adequate. The indicators “complete bladder emptying” and “urinary retention” can be assessed more consistently, allowing for better decision-making^(5)^.

As in the case presented in the previous section, the ND then characterized is “Impaired urinary elimination (00016)”, culminating in the same nursing outcome “Urinary elimination (0503)” and the intervention “Bladder catheterization (0580)”^(3,4,5)^.Once the catheterization is performed, the indicators assessed in the follow-up phase show the same improvement as presented in Chart 4.

It is important to emphasize that USG as an intervention modifies the results achieved. In this hypothetical case, the improvement in outcomes occurred indirectly, by modifying the care plan and identifying the need for bladder catheterization^(4)^.

In this case, assuming the USG showed that the patient’s bladder was empty, the “amount of urine” indicator would remain at score 1; the indicator “empties bladder completely” would not be evaluated, as the patient does not have intravesical contents to be emptied; the “urinary retention” indicator, which was scored as 1, would be scored as 5, as there would be no urine retained in the bladder. Therefore, bladder catheterization would not be performed^(5)^.

In this regard, in the case of bladder USG, there is an important benefit in finding a “non-occurrence” or a “negative test,” preventing the patient from undergoing unnecessary bladder catheterization. Studies show that the use of USG by nurses can indicate the need for bladder catheterization, reducing the occurrence of possible complications from this procedure, especially urinary tract infection^(10,11)^.

## DISCUSSION

The applications of USG in nursing practice are diverse and can be interpreted in both stages of the NP. Furthermore, this “double fitting” is not unprecedented, as it also occurs with other nursing actions^(4)^. The reality is more complex than the five steps involved in the ADPIE model; nursing practice is dynamic, and the processes of reasoning, decision-making, and action occur simultaneously, employing varied resources and thought processes.

A model is a theoretical or real construct that represents aspects of the real world or allows one to reason about that world^(12)^. Yet what makes the ADPIE model representative of PE? Every model is an idealized representation of a target, which is what it intends to represent. Because it is idealized, this representation does not need to be absolute; that is, the model only needs to match the target in some key aspects^(12)^.

In this sense, although the ADPIE model does not represent NP exactly, it provides opportunities for knowledge to be acquired and produced. In a real-world care setting, the NP does not exist as structured, sequential, interdependent, ordered, cyclical, gradual, and linear steps, but it can be explained in that way. In the healthcare setting, nurses assess the patient while simultaneously intervening and evaluating outcomes, generating new diagnoses in a dynamic, rapid, and objective manner^(12,13,14)^.

NANDA-I describes that, through repetition and familiarity with the context and clinical situations experienced, an experienced nurse can quickly move through a stage of the NP^(3)^. This occurs because experienced nurses tend to use non-analytical reasoning, through pattern recognition, which leads to faster decision-making^(15)^. Nursing practice demands relational thinking to understand the complex web of information surrounding patients’ problems^(4,13)^.

In the hypothetical case mentioned above, this potential representational limitation of the ADPIE model appears when it indicates that USG is a monitoring intervention. This occurs because, when conducting a monitoring intervention, if an event occurs that represents a change in the ND, the subsequent steps (evaluation and assessment) may not be necessary, giving the impression that they will not be developed.

When a bladder ultrasound is performed and a high volume of urine retained in the bladder is found, this confirms the occurrence of the indicator “urinary retention,” which is a defining characteristic of the ND “Impaired urinary elimination (00016)”^(3)^. In other words, skipping directly from the implementation phase to the diagnostic phase. This represents non-analytical reasoning which, upon recognizing a pattern, automatically structures the connections between occurrences in reality based on prior experience^(15)^.

This potential limitation of the ADPIE model is overcome after a little reflection. When an USG provides an opportunity for findings, the nursing outcome is automatically identified and evaluated, allowing for a diagnosis focused on the problem. However fast it may seem, the steps take place. Therefore, it is possible to state that the representativeness problem of the ADPIE model is not necessarily that.

The main problem with the ADPIE model and the USG presented here is representational ambiguity, a problem of model accuracy. This is a condition explored in scientific modeling. It occurs because, as already mentioned, models are not absolute representations of their targets, and may lead to inaccurate representations in some aspects^(12)^. This does not render a model useless, it merely presents a limitation in a given situation analyzed^(12)^.

What happens in the case described here is that the reality represented is the same in both arguments, the same hypothetical patient, the same clinical situation, but with two possible representations according to the ADPIE model. In the mental exercises developed, whether considering USG as a component of stage A or stages P and I, the model performs adequately. One reality, two possible representations. Moreover, in cases where USG is considered a nursing intervention, there is a need to repeat the process more often. This can be corrected by moving away from the initial assumption of analyzing a clinical situation in isolation and incorporating other diagnoses, interventions, and outcomes into the care plan.

Additionally, when considering USG as a component of the assessment phase or as an intervention, in both cases there are possible outcomes that need to be discussed. Taking one side or the other in this discussion demands action or triggers a cascade of questions that have to be answered to maintain the theoretical robustness of the NP using SNLS.

In the first case, assuming that USG is not an intervention could mean assuming that other monitoring interventions described in the NIC are also not interventions. These interventions do not modify related or risk factors for NDs, but they support and increase diagnostic accuracy, which allows these actions to be questioned for not having a direct therapeutic effect. This could trigger a broad and complex review of the entire NIC.

In the second case, by assuming that USG is a nursing intervention, it is assumed that it is a “treatment,” as described in the NIC definition of intervention^(4)^. In this sense, it may be necessary to better define the term “treatment” in the NIC or even develop a categorization of nursing interventions according to their objective, including monitoring interventions. It is also possible to suggest a change in the general definition of nursing intervention in the NIC, to include monitoring interventions.

Even in this case, there is a potentially problematic outcome that can occur when assuming that ultrasonography is an intervention, referred to here as “interventionism”. This means that other actions used in clinical trials may also be considered interventions, such as auscultation, percussion, and palpation. Just as USG can be preceded and supported by clinical reasoning, pulmonary auscultation, for example, can also be performed in the same way.

Furthermore, the reflection presented also points to a possible impact on nursing education, which deserves attention. The training of nurses in USG is still in its early stages in Brazil. COFEN, for example, determines the need for training, but does not specify how this training process should occur^(1)^.

Thus, it can be conjectured that by considering USG as an integral part of the nursing physical examination, and therefore as a semiological tool used in the assessment stage, it can be situated outside the exclusive realm of specialties. This approach suggests its inclusion as early as initial training, through subjects such as “fundamentals of nursing,” even during undergraduate studies. On the other hand, when interpreted as an intervention, USG can be understood from a specialized perspective, which reinforces the need for specific and qualified training, considering its operator-dependent nature and requiring an appropriate learning curve.

The inclusion of USG in the NP, based on the ADPIE model, reveals not only the complexity of nursing practice but also the need for critical analysis of the theoretical models that guide it. The ADPIE model, while useful for organizing and streamlining the NP, has limitations when attempting to represent the dynamics of care in its entirety. The representational ambiguity exposes the model’s limitations in capturing the fluidity and simultaneity of nursing actions.

Therefore, far from concluding the discussion, this essay seeks to broaden the debate on how innovative practices, such as USG performed by nurses, are conceptually articulated within the scope of nursing practice. Rather than defining whether it is an intervention or an assessment tool, the aim here is to recognize that nursing practice challenges theoretical models by presenting complex situations that demand flexible and critical interpretations. This tension between practice and theory does not represent a weakness: it is precisely through this confrontation that the science of nursing renews itself, expands its limits, and affirms its relevance in the face of contemporary transformations in care.

## CONCLUSION

This essay demonstrated that incorporating USG into nursing practice challenges theoretical paradigms of the NP, revealing an important duality: USG can act both as an assessment tool and as an intervention. This ambiguity exposes the limitations of the ADPIE model in capturing the non-linear and relational dynamics of real clinical care, especially in situations that require rapid thinking.

As implications, this study points to possible revisions in nursing classification systems, particularly in the NIC, to better accommodate monitoring interventions. In parallel, although ADPIE retains its usefulness as a theoretical framework, its application requires flexibility for non-linear decision-making approaches. To further this discussion, future research could empirically investigate the behavior of USG in NP.

## Data Availability

The entire dataset supporting the results of this study was published in the article itself.
