# Supplementary material for: In which stage of the nursing process is ultrasonography used?
Source: Rev Esc Enferm USP. 2026 Mar 27;60:e20250296. doi: 10.1590/1980-220X-REEUSP-2025-0296en (PMC13041525; doi:10.1590/1980-220X-REEUSP-2025-0296en)
Supplement: Translation

# Translation: Em qual etapa do processo de enfermagem a ultrassonografia se insere?

**DOI:** 10.1590/1980-220X-REEUSP-2025-0296pt

**Authors:** Vítor Monteiro Moraes, Taline Bavaresco, Miriam de Abreu Almeida, Daniele Delacanal Lazzari

**Affiliations:** 1 Universidade Federal de Santa Catarina, Programa de Pós-graduação em Enfermagem, Florianópolis, SC, Brasil.; 2 Universidade Federal do Rio Grande do Sul, Programa de Pós-graduação em Enfermagem, Porto Alegre, RS, Brasil.

**Keywords:** Processo de Enfermagem, Ultrassonografia, Terminologia Padronizada em Enfermagem, Modelos de Enfermagem, Cuidados de Enfermagem

## Abstract

**Objetivo:**

Refletir sobre a inserção da ultrassonografia nas etapas do Processo de Enfermagem.

**Método:**

Ensaio teórico-reflexivo que explora as relações da ultrassonografia com o Processo de Enfermagem, tendo como base os sistemas de linguagem padronizada em enfermagem NANDA *International, Nursing Interventions Classification* e *Nursing Outcomes Classification* e o modelo com cinco etapas do Processo de Enfermagem (Avaliação, Diagnóstico, Planejamento, Implementação e Evolução).

**Resultados:**

Um caso clínico hipotético é utilizado como fio condutor da reflexão, apresentando duas linhas argumentativas: a ultrassonografia incluída na etapa de avaliação (exame físico), ou nas etapas planejamento e implementação do Processo de Enfermagem. É demonstrada uma ambiguidade representacional do modelo teórico com cinco etapas e são discutidas possibilidades e consequências a partir da assunção da ultrassonografia em uma ou outra etapa do Processo de Enfermagem.

**Considerações finais:**

A ultrassonografia desafia paradigmas teóricos do Processo de Enfermagem, não havendo uma posição final sobre em qual etapa melhor se insere. Possíveis refinamentos em sistemas de linguagem padronizada de enfermagem são aventadas e outros estudos sobre a temática são sugeridos.

## INTRODUÇÃO

A ultrassonografia (USG) realizada por enfermeiros foi normatizada pelo Conselho Federal de Enfermagem do Brasil (COFEN) em 2021^(1)^ e preconiza-se que seja realizada por enfermeiros devidamente treinados, vedando a emissão de laudo ou diagnóstico nosológico; à beira do leito ou em ambientes pré-hospitalares, visando orientar procedimentos e identificar fenômenos sensíveis à enfermagem. Desta forma, o uso da USG deve ocorrer no contexto do Processo de Enfermagem (PE)^(1,2)^.

Em 2024, o COFEN atualizou a resolução que trata sobre o desenvolvimento do PE, ratificando o modelo de cinco etapas: Avaliação, Diagnóstico, Planejamento, Implementação e Evolução – modelo ADPIE^(2)^. Os diagnósticos de enfermagem (DE), os resultados e os indicadores, as intervenções e atividades de enfermagem podem ser apoiadas nos Sistemas de Linguagem Padronizada de Enfermagem (SLP) e em protocolos institucionais^(2)^. Dentre estes, destacam-se a Classificação de Diagnósticos de Enfermagem da NANDA *International* (NANDA-I), *Nursing Interventions Classification* (NIC) e a *Nursing Outcomes Classification* (NOC)^(3,4,5)^.

Contudo, a introdução dessa tecnologia na rotina clínica desafia as fronteiras conceituais do próprio PE. Se, por um lado, a USG fornece dados objetivos que qualificam a etapa de avaliação, por outro, quando empregada com base em julgamento clínico prévio para orientar condutas, aproxima-se de uma intervenção de Enfermagem. Tal ambiguidade teórico-prática suscita uma reflexão necessária: a USG à beira leito é um meio de avaliação ou configura, em si, uma intervenção de enfermagem?

Refletir sobre essa questão é inovador por dois motivos principais. Primeiro, porque reposiciona a USG dentro do campo teórico da Enfermagem, testando os limites de modelos consagrados, tal como o ADPIE, e evidenciando possíveis lacunas de representação da prática real. Segundo, porque provoca a reinterpretação das classificações padronizadas (NANDA-I, NIC, NOC), sugerindo que novas categorias ou refinamentos possam ser necessários para contemplar práticas emergentes.

Assim, este ensaio teórico-reflexivo tem por objetivo refletir sobre a inserção da USG nas etapas do PE à luz dos SLP em enfermagem e do modelo teórico ADPIE. Por meio de uma análise reflexiva, busca-se desvelar as implicações dessa inserção para o desenvolvimento da ciência de enfermagem e ampliar o debate sobre inovação tecnológica e epistemologia do cuidado. Parte-se do entendimento de que as práticas de cuidado contemporâneas, embora devam se ancorar em modelos teóricos que as suportem, incluam e expliquem no processo de cuidado, frequentemente transcendem os limites rígidos dos arcabouços teóricos tradicionais.

## MÉTODO

Trata-se de um ensaio teórico-reflexivo estruturado para responder à pergunta: *a USG realizada por enfermeiros, no âmbito do PE, deve ser compreendida como componente da etapa de avaliação ou como intervenção de enfermagem?*

### Referencial Teórico-Metodológico

A reflexão foi ancorada no modelo de organização do PE (ADPIE) como estrutura lógico-procedimental do cuidado; nos SLP em enfermagem (NANDA-I, NIC, NOC), como ontologias operacionais que vinculam diagnóstico-resultado-intervenção; na literatura de representação/modelagem científica, para discutir limites e acurácia representacional de modelos quando confrontados com a prática; e aportes sobre raciocínio clínico em enfermagem (analítico vs. não-analítico/por padrões), para explicitar como decisões à beira-leito podem tensionar fronteiras conceituais entre avaliar e intervir.

### Corpus Teórico

O *corpus* reuniu: (a) normativas profissionais pertinentes à USG por enfermeiros e ao desenvolvimento obrigatório do PE; (b) edições vigentes das classificações NANDA-I, NIC e NOC; (c) publicações sobre USG no cuidado de enfermagem/POCUS e sobre desenvolvimento/validação de intervenções de monitoração; (d) obras de referência em representação científica e raciocínio clínico. A suficiência do *corpus* foi guiada por saturação teórica referente à pergunta norteadora.

### Estratégia Analítica

A análise combinou: (1) análise conceitual (esclarecimento de termos e relações entre categorias NANDA-I/NIC/NOC no contexto do ADPIE); (2) contraste de cenários (USG como extensão da avaliação versus USG como intervenção de enfermagem, com foco em intervenções de monitoração); (3) um experimento mental com caso clínico hipotético em terapia intensiva, utilizado como “dispositivo de prova” para testar a performance explicativa do modelo ADPIE sob cada enquadramento; e (4) análise de consequências (implicações de cada enquadramento para ensino, prática clínica e possíveis ajustes nas classificações).

### Construção e Uso do Caso Hipotético

O caso foi construído a partir de situações frequentes em prática hospitalar (pós-operatório, risco de retenção urinária, decisão sobre cateterismo vesical). Foram explicitados dados clínicos, diagnósticos de enfermagem elegíveis, resultados esperados e intervenções possíveis. A USG foi incorporada como passo decisório para demonstrar, comparativamente, como o enquadramento, a avaliação ou a intervenção altera o fluxo lógico do PE, a valoração de indicadores da NOC e a seleção de intervenções na NIC.

### Critérios de Rigor e Credibilidade

Para assegurar qualidade teórica, adotaram-se: coerência interna do argumento; transparência na cadeia de inferências; auditabilidade das fontes (citações diretas às classificações e normativas); triangulação conceitual entre ADPIE, NANDA-I/NIC/NOC e literatura de modelagem científica; e busca deliberada de contraexemplos (p.ex., situações em que a USG produz “efeito indireto” sobre resultados).

### Aspectos Éticos

Por se tratar de um ensaio de caráter teórico-reflexivo, esta pesquisa não foi submetida à apreciação de um comitê de ética. O caso clínico utilizado para ilustração é hipotético e não se refere a nenhum paciente real. Ademais, respeitam-se rigorosamente as boas práticas acadêmicas para a produção do conhecimento, assegurando a citação correta e a referência adequada de todas as fontes utilizadas.

## A ULTRASSONOGRAFIA FAZ PARTE DO EXAME FÍSICO DE ENFERMAGEM, COMPONDO A ETAPA DE AVALIAÇÃO DO PACIENTE À BEIRA-LEITO?

A USG permite visualizar e interpretar estruturas do corpo humano, caracterizando-as e permitindo inferências sobre seu funcionamento. Isso, per se, caracteriza-se como uma extensão do exame físico^(6)^. A avaliação de enfermagem pode ser realizada com uso de instrumentos, inclusive exames de imagem^(2)^. A NANDA-I descreve de forma sistematizada e estruturada todo percurso de raciocínio desde a avaliação até a identificação do DE prioritário, podendo-se incluir a USG na avaliação de rastreamento e avaliação aprofundada^(3)^.

A avaliação de rastreamento é considerada a fase inicial da avaliação, em que os primeiros dados são coletados e incitam o início do raciocínio clínico. Portanto, a USG pode enriquecer esse momento enquanto artefato que apoia e qualifica o raciocínio clínico^(3)^. A partir dos dados de rastreamento é que se direciona e se aprofunda o processo de avaliação, resultando em um DE^(3)^. Se a USG é realizada em resposta a uma necessidade percebida no rastreamento, esta pode trazer informações objetivas que direcionam a DE específicos. Isso também possibilita perceber que a NANDA-I preconiza o pensamento hipotético-dedutivo, ao prever a utilização de procedimentos específicos de coleta de dados para confirmar ou refutar hipóteses diagnósticas^(3)^.

Além dos argumentos já colocados, é importante compreender como a USG se insere no modelo ADPIE nessa linha argumentativa. Em um caso hipotético, um enfermeiro chega à Unidade de Terapia Intensiva (UTI) às 13 horas para um turno de seis horas. Recebe o caso de um paciente masculino, 80 anos, que realizou cirurgia abdominal, está restrito ao leito e possui como comorbidade Hiperplasia Prostática Benigna (HPB). Ao realizar a avaliação do paciente, o enfermeiro percebe que o cateter vesical de demora (CVD) foi retirado, encontrando nos registros de enfermagem que isso ocorreu às 12 horas (letra A do modelo ADPIE). Definiu-se o DE “Risco de Retenção Urinária (00322)”, cujos fatores de risco nesse caso são “postura inadequada no vaso sanitário” e “privacidade inadequada”, e como condição associada, “doenças prostáticas”. Isso compõe a etapa de diagnóstico, letra D^(3)^.

Na etapa de planejamento (letra P), o enfermeiro elenca o resultado da NOC “Eliminação Urinária (0503)” e a intervenção “Assistência no autocuidado: uso do vaso sanitário (1804)” da NIC^(4,5)^. Esta intervenção inclui auxílio na eliminação urinária, fornecimento de dispositivos auxiliares quando necessário e garantia de privacidade. Os indicadores clínicos valorados em escala Likert de 5 pontos, em que quanto maior a pontuação, mais desejável é o estado, estão descritos no Quadro 1 ^(5)^.

**Quadro 1:** Indicadores do resultado de Enfermagem “Eliminação Urinária (0503)” escolhidos para o caso hipotético – Florianópolis, SC, Brasil, 2025.

Indicador	Escala de mensuração				
	1. Gravemente comprometido	2. Muito comprometido	3. Moderadamente comprometido	4. Levemente comprometido	5. Não comprometido
Reconhecimento da vontade					X O
Padrão de eliminação					X O
Quantidade de urina					X O
Esvazia a bexiga completamente					X O
	1 Grave	2 Substancial	3 Moderado	4 Leve	5 Nenhum
Retenção urinária					X O

Nota: “X” representa o estado atual do paciente e o “O”, o estado esperado. Fonte: Adaptado de Moorhead, Johnson e Swanson (2024, p. 408–409)^(5)^.

A implementação do plano de cuidados ao longo do turno do enfermeiro neste caso hipotético é considerada como concluída, uma vez que as atividades de cuidado foram desenvolvidas sem particularidades dignas de nota. Assim, compõe-se a etapa I.

A evolução de enfermagem, etapa seguinte e final do PE é descrita como a “avaliação dos resultados alcançados de enfermagem e saúde da pessoa, família, coletividade e grupos especiais”^(2)^, e considera que esta etapa “permite a análise e a revisão de todo o Processo de Enfermagem”^(2)^. É na etapa de evolução que os indicadores dos resultados de enfermagem são valorados e analisado o comportamento desses indicadores a partir do plano de cuidados que foi executado^(2,5)^ (Quadro 2).

**Quadro 2:** Escores alcançados nos indicadores do resultado de enfermagem “Eliminação Urinária (0503)” valorados às 18 horas – Florianópolis, SC, Brasil, 2025.

Indicador	Escala de mensuração					
	1. Gravemente comprometido	2. Muito comprometido	3. Moderadamente comprometido	4. Levemente comprometido	5. Não comprometido	NA*
Reconhecimento da vontade	X					
Padrão de eliminação	X					
Quantidade de urina						X
Esvazia a bexiga completamente	X					
	1 Grave	2 Substancial	3 Moderado	4 Leve	5 Nenhum	
Retenção urinária	X					

Nota: NA*: Não avaliado. “X” representa o escore alcançado. Fonte: Adaptado de Moorhead, Johnson e Swanson (2024, p.408–409)^(5)^.

Na avaliação das 18h, o paciente apresentou escore 1 em “reconhecimento da vontade” pela ausência de desejo miccional e “padrão de eliminação” pois não apresentou nenhuma diurese em seis horas^(5)^. A “quantidade de urina” não foi avaliada pois o paciente não urinou e a avaliação da região suprapúbica com palpação e percussão fica prejudicada pela ferida operatória abdominal. O fato de o paciente não ter esvaziado a bexiga e, ao ser oferecido urinol, referiu que não conseguia urinar, justifica o escore 1 nos indicadores “esvazia a bexiga completamente” e “retenção urinária”^(5)^.

O enfermeiro então decide avaliar o paciente com USG. Quando isso ocorre, está recomeçando o PE, retomando a primeira etapa do ADPIE. Ao realizar a USG de bexiga constata que há quantidade importante de urina retida na bexiga. Isso permite identificar a forma que a USG se relaciona com a etapa de “avaliação aprofundada” pela NANDA-I, ao subsidiar o raciocínio clínico e diagnóstico^(3)^.

Assim, o DE muda para “Eliminação urinária prejudicada (00016)”, que é definido como “inabilidade de excretar de forma efetiva fluidos e resíduos armazenados na bexiga pela uretra”^(3)^. A característica definidora encontrada é “retenção urinária”, tendo como fatores relacionados “postura inadequada no vaso sanitário” e “privacidade inadequada”^(3)^. O paciente encontra-se ainda em uma população em risco, por ser idoso, e tem como condição associada a HPB^(3)^.

Na etapa de planejamento, o enfermeiro revisita o quadro clínico para identificar o estado atual do paciente e elencar intervenções que permitam alcançar os resultados esperados^(2)^. O estado atual do paciente é o mesmo já apresentado no Quadro 2, acrescido dos achados oportunizados pela USG (Quadro 3).

**Quadro 3:** Etapa de planejamento após avaliação com USG de bexiga – Florianópolis, SC, Brasil, 2025.

Indicador	Escala de mensuração				
	1. Gravemente comprometido	2. Muito comprometido	3. Moderadamente comprometido	4. Levemente comprometido	5. Não comprometido
Reconhecimento da vontade	X				O
Padrão de eliminação	X				O
Quantidade de urina					X O
Esvazia a bexiga completamente	X				O
	1 Grave	2 Substancial	3 Moderado	4 Leve	5 Nenhum
Retenção urinária	X				O

Nota: “X” representa o estado atual do paciente e o “O”, o estado esperado. Fonte: Adaptado de Moorhead, Johnson e Swanson (2024, p.408–409)^(5)^.

Houve uma mudança nos indicadores avaliados após aplicação da USG no caso. O indicador “quantidade de urina”, que não havia sido avaliado, passa a constar como escore 5 e isso ocorreu pois a USG oportunizou a mensuração do volume vesical de forma não invasiva. Além disso, permitiu avaliar de forma mais consistente os indicadores “esvazia a bexiga completamente” e “retenção urinária”^(5)^.

Dessa forma, o enfermeiro decide realizar a intervenção “Cateterismo vesical (0580)”, direcionando a intervenção à característica definidora “retenção urinária” e não mais aos fatores relacionados^(3,4)^. O volume de urina drenado foi de 550 mililitros. A partir disso é possível reavaliar os escores alcançados em cada indicador clínico do resultado “Eliminação urinária (0503)”^5^ (Quadro 4).

**Quadro 4:** Escores alcançados nos indicadores clínicos avaliados no caso após realização de cateterismo vesical – Florianópolis, SC, Brasil, 2025.

Indicador	Escala de mensuração				
	1. Gravemente comprometido	2. Muito comprometido	3. Moderadamente comprometido	4. Levemente comprometido	5. Não comprometido
Reconhecimento da vontade	X				
Padrão de eliminação			X		
Quantidade de urina					X
Esvazia a bexiga completamente					X
	1 Grave	2 Substancial	3 Moderado	4 Leve	5 Nenhum
Retenção urinária					X

Nota: “X” representa o escore alcançado. Fonte: Adaptado de Moorhead, Johnson e Swanson (2024, p.408–409)^(5)^.

Nesse ponto, novamente na etapa E do modelo ADPIE, são cotejados os resultados obtidos. O indicador “reconhecimento da vontade” segue como escore 1, pois o paciente apresentou urina retida na bexiga e não referiu vontade de urinar ou desconforto. O “padrão de eliminação” é parcialmente restabelecido a partir do cateterismo, avaliado como moderadamente comprometido (escore 3). O indicador “quantidade de urina” não foi modificado, pois a USG demonstrou quantidade adequada de urina, o que foi ratificado com o cateterismo. Já os indicadores “esvazia a bexiga completamente” e “retenção urinária” são plenamente resolvidos a partir da intervenção realizada. Isso finda a descrição do exercício mental com aplicação da USG no modelo ADPIE na primeira etapa do PE, a avaliação^(5)^.

O caso demonstra que a USG se encaixa na etapa de avaliação. Além disso, a USG não atende à definição de intervenção de enfermagem da NIC, que é “qualquer tratamento que, baseado em julgamento e conhecimento clínico, um enfermeiro ponha em prática para melhorar os resultados do paciente”^(4)^. Nesse sentido, a USG não é um “tratamento”, pois não modificou diretamente os resultados de enfermagem.

## A ULTRASSONOGRAFIA É UMA INTERVENÇÃO DE ENFERMAGEM?

A NIC considera, desde 1996, que a USG realizada por enfermeiros é uma intervenção de enfermagem: “Ultrassonografia: obstétrica e ginecológica (6982)”^(4)^. Atualmente, na oitava edição, há uma segunda intervenção de enfermagem com uso de USG: “Ultrassonografia: bexiga (0565)”^(4,5,6,7)^. Esta última foi desenvolvida recentemente^(7)^ e é definida como “realização de exames por ultrassonografia para determinar a função ou a estrutura da bexiga”^(4)^.

Somando à definição da NIC para intervenção de enfermagem, a NANDA-I traz, ao descrever o Modelo Tripartite de Kamitsuru, que “as ações ou intervenções autônomas de enfermagem são *tratamentos* baseados em padrões de conhecimento de enfermagem, determinados pelo enfermeiro como adequados para abordar os fatores etiológicos de um DE ou manejar sintomas”^(3)^ (grifo nosso). Na presente linha argumentativa, a USG pode ser considerada um tratamento, por ser direcionada ao DE e melhorar os resultados.

O termo “tratamento” não é formalmente descrito na NIC ou na classificação da NANDA-I. No Dicionário Online de Português, um dos significados do verbete “tratamento” é o “conjunto de meios práticos para combater uma doença; terapêutica”^(8)^. Já o Dicionário Michaelis, também online, traz como um dos significados desse mesmo verbete “conjunto de meios de que lança mão o médico para cura ou alívio do doente”^(9)^.

Em comum, os significados linguísticos do termo “tratamento” trazem o “conjunto de meios”. Nesse sentido, a USG realizada por enfermeiros compõe o “conjunto de meios”, e é, em si, também um “conjunto de meios” ou ações que permitem a obtenção de melhores resultados, sendo este o seu “efeito terapêutico”, que lhe denota a ideia de “tratamento”. É importante destacar também que esse “efeito”, nesse caso, não é direto, mas sim indireto. Isso ocorre pois as intervenções com uso de USG podem ser consideradas como intervenções de monitoração, conforme descrito na NIC^(4)^.

A NIC inclui inúmeras intervenções de monitoração, tais como monitoração ácido básica e dos sinais vitais, que incorporam e refletem o julgamento clínico e indicam ao enfermeiro o que deve procurar e o que fazer quando se estabelece algum evento^(4)^.

Além disso, é importante destacar que diagnósticos de risco demandam intervenções de monitoração. A NANDA-I define os DE de risco como a suscetibilidade de desenvolver, no futuro, uma resposta humana indesejável^(3)^. Além de agir para controlar os fatores de risco, também é necessário que seja identificada a ocorrência do evento em risco. Em um exemplo rápido, o diagnóstico “Risco de lesão por pressão no adulto (00304)” pode ser modificado ou atenuado diretamente pela intervenção “Prevenção de lesões por pressão (3540)” a partir do controle de fatores de risco, e a ocorrência do diagnóstico com foco no problema – “Lesão por pressão no adulto (00312)” – pode ser verificada pela intervenção de monitoração “Supervisão da pele (3590)”^(3,4)^.

Exemplificando, para que se realize a intervenção “Monitoração de sinais vitais (6680)”, é necessário que se tenha o domínio de técnicas tais como aferição de pressão arterial, saturação periférica de oxigênio, temperatura corporal, frequência cardíaca, etc^(4)^. Esse “saber-fazer” é o que delimita a intervenção. Esta é uma intervenção precedida de raciocínio clínico que a embasa e a provoca no plano assistencial para obtenção de resultados específicos^(4)^.

Contrariamente à seção anterior, será realizado o exercício mental de encaixar a USG no modelo ADPIE, dessa vez como intervenção de enfermagem, utilizando o mesmo caso hipotético. Então, com o DE de “Risco de Retenção Urinária (00322)”, NOC “Eliminação Urinária (0503)” e NIC “Assistência no autocuidado: uso do vaso sanitário (1804)”^(3,4,5)^, às 18 horas do dia em questão, o paciente apresenta os escores avaliados já descritos no Quadro 2.

Ao avaliar esses escores, o enfermeiro constata que o DE do paciente se mantém o mesmo, “Risco de Retenção Urinária (00322)”^(3)^. E isso porque é possível julgar que não há indícios suficientes que demandem sua mudança, uma vez que novos fatores de risco não ocorreram e os já existentes não foram resolvidos. Além disso, a ocorrência de retenção urinária pode ser mais bem avaliada com o uso da intervenção de enfermagem “Ultrassonografia: bexiga (0565)”, pois assim como é possível o paciente estar apresentando “Eliminação Urinária Prejudicada (00016)”, também é possível que tenha havido redução na produção de urina^(3,4)^. A diferença é que, em um caso, a realização do cateterismo vesical é uma intervenção que pode ser aventada; no outro, não.

Dessa forma, no alcance do resultado “Eliminação Urinária (0503)”, o enfermeiro elenca a intervenção de enfermagem “Ultrassonografia: bexiga (0565)”. Nessa etapa de planejamento, poderia também ser elencada a intervenção “Cateterismo vesical (0580)” a ser realizada a depender do resultado alcançado pela intervenção “Ultrassonografia: bexiga (0565)”^(4,5)^. Propõe-se, portanto, neste exercício, neste momento, a realização apenas da intervenção “Ultrassonografia: bexiga (0565)”^(4)^ (Quadro 5).

**Quadro 5:** Estado atual do paciente antes da realização da intervenção de enfermagem “Ultrassonografia: bexiga (0565)” e o estado esperado –Florianópolis, SC, Brasil, 2025.

Indicador	Escala de mensuração					
	1. Gravemente comprometido	2. Muito comprometido	3. Moderadamente comprometido	4. Levemente comprometido	5. Não comprometido	NA*
Reconhecimento da vontade	X				O	
Padrão de eliminação	X				O	
Quantidade de urina					O	X
Esvazia a bexiga completamente	X				O	
	1 Grave	2 Substancial	3 Moderado	4 Leve	5 Nenhum	NA*
Retenção urinária	X				O	

Nota: NA*: Não avaliado. “X” representa o estado atual do paciente e o “O”, o estado esperado. Fonte: Adaptado de Moorhead, Johnson e Swanson (2024, p.408–409)^(5)^.

Diferente da linha argumentativa anterior, percebe-se que o estado atual é o mesmo da etapa de evolução do ciclo que iniciou o presente exercício. A etapa de implementação ocorre pela realização da intervenção “Ultrassonografia: bexiga (0565)”^(4)^. Ao realizar a intervenção o enfermeiro identificou um volume urinário aumentado a partir da USG. Assim, o resultado de enfermagem pode novamente ser valorado (Quadro 6).

**Quadro 6:** Escores alcançados pelo paciente após a realização da intervenção de enfermagem “Ultrassonografia: bexiga (0565)” – Florianópolis, SC, Brasil, 2025.

Indicador	Escala de mensuração					
	1. Gravemente comprometido	2. Muito comprometido	3. Moderadamente comprometido	4. Levemente comprometido	5. Não comprometido	NA*
Reconhecimento da vontade	X					
Padrão de eliminação	X					
Quantidade de urina					X	
Esvazia a bexiga completamente	X					
	1 Grave	2 Substancial	3 Moderado	4 Leve	5 Nenhum	NA*
Retenção urinária	X					

Nota: NA*: Não avaliado. “X” representa o escore alcançado. Fonte: Adaptado de Moorhead, Johnson e Swanson (2024, p. 408–409)^(5)^.

A partir dos escores alcançados é possível identificar que o indicador “quantidade de urina” após realização da USG pode ser avaliado e é adequado. Os indicadores “esvazia a bexiga completamente” e “retenção urinária” podem ser avaliados de forma mais consistente, oportunizando uma melhor tomada de decisão^(5)^.

Assim como no caso apresentado na seção anterior, o DE caracterizado então é “Eliminação urinária prejudicada (00016)”, culminando no mesmo resultado de enfermagem “Eliminação urinária (0503)” e a intervenção “Cateterismo vesical (0580)”^(3,4,5)^. Uma vez realizado o cateterismo, os indicadores valorados na etapa de evolução apresentam a mesma melhora apresentada no Quadro 4.

É importante reforçar que a USG enquanto intervenção modifica os resultados alcançados. No caso hipotético, a melhoria dos resultados ocorreu de forma indireta, ao modificar o plano de cuidados e identificar a necessidade do cateterismo vesical^(4)^.

Nesse caso, supondo que a USG demonstrasse que a bexiga do paciente estava vazia, o indicador “quantidade de urina” seria mantido no escore 1; o indicador “esvazia a bexiga completamente” não seria avaliado, pois o paciente não possui conteúdo intravesical para que seja esvaziado; o indicador “retenção urinária” que estava pontuado como escore 1, seria pontuado como escore 5, pois não haveria urina retida na bexiga. Dessa forma, não seria realizado o cateterismo vesical^(5)^.

Nesse sentido, no caso da USG de bexiga, há um benefício importante na constatação de “não-ocorrência” ou no “teste negativo”, evitando que o paciente seja submetido ao cateterismo vesical de forma desnecessária. Estudos demonstram que a utilização da USG por enfermeiros é capaz de indicar a necessidade de cateterismo vesical e isso reduz a ocorrência das complicações possíveis desse procedimento, especialmente a infecção do trato urinário^(10,11)^.

## DISCUSSÃO

As aplicações da USG na prática de enfermagem são diversas e passíveis de serem interpretadas em ambas as etapas do PE. Além disso, esse “duplo encaixe” não é algo inédito, pois também ocorre com outras ações de enfermagem^(4)^. A realidade é mais complexa que as cinco etapas que envolvem o modelo ADPIE, a prática de enfermagem é dinâmica e os processos de raciocínio, tomada de decisão e de ação ocorrem de forma simultânea e com variados recursos e percursos de pensamento empregados.

Um modelo é um construto teórico ou real que representa aspectos do mundo real ou permite que se raciocine sobre esse mundo^(12)^. Mas o que faz com que o modelo ADPIE represente o PE? Todo modelo é uma representação idealizada de um alvo, que é o que se pretende representar. Por ser idealizada, essa representação não necessita ser absoluta, ou seja, o modelo precisa corresponder ao alvo apenas em alguns aspectos chave^(12)^.

Nesse sentido, embora o modelo ADPIE não represente o PE de forma exata, oportuniza que conhecimentos sejam obtidos e produzidos. No ambiente real de cuidado o PE não existe enquanto etapas estruturadas, sequenciais, interdependentes, ordenadas, cíclicas, graduais e lineares, mas pode ser explicado dessa maneira. No cenário assistencial, os enfermeiros avaliam o paciente, ao mesmo tempo que intervêm e avaliam resultados, gerando simultaneamente novos diagnósticos, de forma dinâmica, rápida e objetiva^(12,13,14)^.

A NANDA-I descreve que, a partir da repetição e familiaridade com o contexto e as situações clínicas vivenciadas, um enfermeiro experiente consegue rapidamente passar por uma etapa do PE^(3)^. Isso ocorre pois enfermeiros experientes tendem a usar o raciocínio não-analítico, por reconhecimento de padrões, que leva a tomadas de decisão mais rápidas^(15)^. A prática de enfermagem demanda pensamento relacional para compreender o complexo emaranhado de informações que envolve os problemas do paciente^(4,13)^.

No caso hipotético supracitado, essa possível limitação representacional do modelo ADPIE aparece quando indica que a USG é uma intervenção de monitoração. Isso ocorre pois, ao realizar uma intervenção de monitoração, quando há ocorrência de um evento que represente a mudança no DE, as etapas subsequentes (evolução e avaliação) podem não ser necessárias, dando a impressão de que não serão desenvolvidas.

Quando realizada a USG de bexiga e constatado o elevado volume urinário retido na bexiga, há a comprovação da ocorrência do indicador “retenção urinária” que é uma característica definidora do DE “Eliminação urinária prejudicada (00016)”^(3)^. Ou seja, pulando da etapa de implementação diretamente para a etapa de diagnóstico. Isso representa o raciocínio não-analítico que, ao reconhecer um padrão, automaticamente estrutura as ligações entre as ocorrências na realidade com base em experiência prévia^(15)^.

Essa possível limitação do modelo ADPIE é superada após pequena reflexão. Quando há o achado oportunizado pela USG, há automática identificação e valoração do resultado de enfermagem, permitindo chegar ao diagnóstico com foco no problema. Por mais rápido que isso possa parecer, as etapas ocorrem. Dessa forma, é possível afirmar que o problema de representatividade do modelo ADPIE não é necessariamente esse.

O principal problema com o modelo ADPIE e a USG apresentado aqui é a ambiguidade representacional, um problema de acurácia do modelo. Essa é uma condição explorada na modelagem científica e ocorre, pois, como já dito, os modelos não são representações absolutas de seus alvos, podendo levar a representações inacuradas em alguns aspectos^(12)^. Isso não inutiliza um modelo, apenas apresenta uma limitação em determinada situação analisada^(12)^.

O que acontece no caso aqui descrito é que a realidade representada é a mesma em ambos os argumentos, o mesmo paciente hipotético, a mesma situação clínica, mas com duas representações possíveis pelo modelo ADPIE. Nos exercícios mentais desenvolvidos, seja considerando a USG como componente da etapa A ou das etapas P e I, o modelo performa adequadamente. Uma realidade, duas representações possíveis. Ainda, no caso em que a USG é considerada intervenção de enfermagem há uma necessidade de girar o processo mais vezes, o que pode ser corrigido deixando de assumir o pressuposto inicial de analisar uma situação clínica isoladamente, incorporando outros diagnósticos, intervenções e resultados no plano de cuidados.

Além disso, ao considerar a USG enquanto componente da etapa de avaliação ou enquanto uma intervenção, em ambos os casos existem desdobramentos possíveis que precisam ser discutidos. Assumir um lado ou outro dessa discussão demanda ações ou desencadeia questionamentos em cascata que precisam ser respondidos para manutenção da robustez teórica do PE com uso de SLP.

No primeiro caso, assumir que a USG não é uma intervenção pode significar assumir que outras intervenções de monitoração descritas na NIC também não são intervenções. Essas intervenções não modificam fatores relacionados ou de risco dos DE, mas subsidiam e aumentam a acurácia diagnóstica, o que permite que estas ações sejam questionadas por não possuírem um efeito terapêutico direto. Isso poderia desencadear uma revisão ampla e complexa de toda NIC.

No segundo caso, ao assumir que a USG é uma intervenção de enfermagem, assume-se que esta é um “tratamento”, conforme é descrito na definição de intervenção da NIC^(4)^. Nesse sentido, pode ser necessário definir melhor na NIC o termo “tratamento” ou ainda desenvolver uma categorização das intervenções de enfermagem de acordo com seu objetivo, contemplando as de monitoração. É possível aventar também uma mudança na definição geral de intervenção de enfermagem da NIC, possibilitando contemplar as intervenções de monitoração.

Ainda nesse caso, há um possível desdobramento problemático que pode ocorrer ao se assumir que a USG é uma intervenção, denominada aqui de “intervencionismo”. Isso significa que é possível que outras ações que são utilizadas enquanto testes clínicos passem também a ser consideradas como intervenção, como é o caso da ausculta, da percussão e da palpação. Da mesma forma que a USG pode ser precedida e subsidiada por raciocínio clínico, a ausculta pulmonar, por exemplo, também pode ocorrer da mesma forma.

Além disso, a reflexão apresentada também aponta para um possível impacto no ensino de enfermagem e que merece atenção. O aprendizado da USG por enfermeiros ainda se apresenta de forma incipiente no Brasil. O COFEN, por exemplo, determina a necessidade de treinamento, mas não qualifica como esse processo formativo deve ocorrer^(1)^.

Assim, pode-se conjecturar que ao considerar a USG como parte integrante do exame físico de enfermagem, portanto, como uma ferramenta semiológica utilizada na etapa de avaliação, ela pode ser situada fora da ordem exclusiva das especialidades. Essa abordagem sugere sua inclusão já na formação inicial, por meio de disciplinas como “fundamentos de enfermagem”, ainda na graduação. Por outro lado, ao ser interpretada como uma intervenção, a USG pode ser compreendida sob uma ótica especializada, o que reforça a necessidade de capacitação específica e qualificada, considerando inclusive sua natureza operador-dependente e demandando curva de aprendizado adequada.

O enquadramento da USG no PE a partir do modelo ADPIE revela não apenas a complexidade da prática de enfermagem, mas também a necessidade de análise crítica sobre os modelos teóricos que a orientam. O modelo ADPIE, apesar de ser útil para organizar e racionalizar o PE, apresenta limitações ao tentar representar a dinâmica do cuidado em sua totalidade. A ambiguidade representacional expõe as limitações do modelo em capturar a fluidez e a simultaneidade das ações de enfermagem.

Portanto, longe de encerrar a discussão, este ensaio busca ampliar o debate sobre como práticas inovadoras, tais como a USG realizada por enfermeiros, se articulam conceitualmente no âmbito do PE. Mais do que definir se se trata de uma intervenção ou de uma ferramenta de avaliação, a proposta aqui é reconhecer que a prática de enfermagem desafia os modelos teóricos, ao apresentar situações complexas que exigem interpretações flexíveis e críticas. Essa tensão entre prática e teoria não representa uma fragilidade: é justamente por meio desse confronto que a ciência da enfermagem se renova, expande seus limites e afirma sua relevância frente às transformações contemporâneas do cuidado.

## CONSIDERAÇÕES FINAIS

Este ensaio demonstrou que a incorporação USG na prática de enfermagem desafia paradigmas teóricos do PE, revelando uma dualidade importante: a USG pode atuar tanto como ferramenta de avaliação quanto como intervenção. Essa ambiguidade expõe as limitações do modelo ADPIE em capturar a dinâmica não linear e relacional do cuidado clínico real, especialmente em situações que exigem raciocínio rápido.

Como implicações, este estudo aponta para possíveis revisões nos sistemas de classificação de enfermagem, particularmente na NIC, para melhor acomodar intervenções de monitoração. Paralelamente, embora o ADPIE mantenha sua utilidade como estrutura teórica, sua aplicação requer flexibilidade para abordagens não lineares de tomada de decisão. Para avançar nessa discussão, pesquisas futuras podem investigar empiricamente o comportamento da USG no PE.

## Data Availability

Todo o conjunto de dados que dá suporte aos resultados deste estudo foi publicado no próprio artigo.

# Translation: ¿En qué etapa del proceso de enfermería se inserta la ultrasonografía?

**DOI:** 10.1590/1980-220X-REEUSP-2025-0296es

**Authors:** Vítor Monteiro Moraes, Taline Bavaresco, Miriam de Abreu Almeida, Daniele Delacanal Lazzari

**Affiliations:** 1 Universidade Federal de Santa Catarina, Programa de Pós-graduação em Enfermagem, Florianópolis, SC, Brasil.; 2 Universidade Federal do Rio Grande do Sul, Programa de Pós-graduação em Enfermagem, Porto Alegre, RS, Brasil.

**Keywords:** Proceso de Enfermería, Ultrassonografía, Terminología Normalizada de Enfermería, Modelos de Enfermería, Atención de Enfermería

## Abstract

**Objetivo:**

Reflexionar sobre la inclusión de la ultrasonografía en las etapas del Proceso de Enfermería.

**Método:**

Ensayo teórico-reflexivo que explora la relación entre la ultrasonografía y el Proceso de Enfermería, basado en los sistemas de lenguaje de enfermería estandarizados NANDA *Internacional, y Nursing Outcomes Classification* y el modelo de cinco pasos del Proceso de Enfermería (Valoración, Diagnóstico, Planificación, Implementación y Evaluación).

**Resultados:**

Se utiliza un caso clínico hipotético como hilo conductor de la reflexión, presentando dos líneas de argumentación: la inclusión de la ultrasonografía en la etapa de valoración (examen físico), o bien en las etapas de planificación e implementación del Proceso de Enfermería. Se demuestra la ambigüedad representacional del modelo teórico de cinco etapas y se discuten las posibilidades y consecuencias de incluir la ecografía en una u otra etapa del Proceso de Enfermería.

**Conclusión:**

La ultrasonografía desafía los paradigmas teóricos del Proceso de Enfermería y no hay una respuesta definitiva sobre en qué etapa encaja mejor. Se discuten posibles mejoras en los sistemas de lenguaje de enfermería estandarizados y se sugieren estudios adicionales sobre el tema.

## INTRODUCCIÓN

La ultrasonografía (USG) realizada por enfermeros fue normalizada por el Consejo Federal de Enfermería de Brasil (COFEN) en 2021^(1)^ y se recomienda que sea realizada por enfermeros debidamente capacitados, prohibiendo la emisión de informes o diagnósticos nosológicos; a pie de cama o en entornos prehospitalarios, con el fin de orientar los procedimientos e identificar fenómenos sensibles a la enfermería. De este modo, el uso de la USG debe producirse en el contexto del Proceso de Enfermería (PE)^(1,2)^.

En 2024, el COFEN actualizó la resolución que trata sobre el desarrollo del PE, ratificando el modelo de cinco etapas: Avaluación, Diagnóstico, Planificación, Implementación y Evolución – modelo ADPIE^(2)^. Los diagnósticos de enfermería (DE), los resultados y los indicadores, las intervenciones y las actividades de enfermería pueden basarse en los Sistemas de Lenguaje Estandarizado de Enfermería (Sistemas de Linguagem Padronizada - SLP) y en los protocolos institucionales^(2)^. Entre ellos, destacan la Clasificación de Diagnósticos de Enfermería de NANDA *International* (NANDA-I), la *Clasificación de Intervenciones de Enfermería (Nursing Interventions Classification* - NIC) y la *Clasificación de Resultados de Enfermería (Nursing Outcomes Classification* - NOC)^(3,4,5)^.

Sin embargo, la introducción de esta tecnología en la rutina clínica desafía los límites conceptuales del propio PE. Si, por un lado, la USG proporciona datos objetivos que califican la etapa de evaluación, por otro, cuando se emplea sobre la base de un juicio clínico previo para orientar las conductas, se aproxima a una intervención de enfermería. Esta ambigüedad teórico-práctica suscita una reflexión necesaria: ¿la USG a pie de cama es un medio de evaluación o constituye, en sí misma, una intervención de enfermería?

Reflexionar sobre esta cuestión es innovador por dos razones principales. En primer lugar, porque reposiciona la USG dentro del campo teórico de la enfermería, poniendo a prueba los límites de modelos consagrados, como el ADPIE, y poniendo de manifiesto posibles lagunas en la representación de la práctica real. En segundo lugar, porque provoca la reinterpretación de las clasificaciones estandarizadas (NANDA-I, NIC, NOC), sugiriendo que pueden ser necesarias nuevas categorías o refinamientos para contemplar prácticas emergentes.

Así, este ensayo teórico-reflexivo tiene como objetivo reflexionar sobre la inserción de la USG en las etapas del PE a la luz de los SLP en enfermería y del modelo teórico ADPIE. A través de un análisis reflexivo, se busca revelar las implicaciones de esta inserción para el desarrollo de la ciencia de la enfermería y ampliar el debate sobre la innovación tecnológica y la epistemología del cuidado. Se parte de la base de que las prácticas de cuidado contemporáneas, aunque deben basarse en modelos teóricos que las respalden, las incluyan y las expliquen en el proceso de cuidado, a menudo trascienden los límites rígidos de los marcos teóricos tradicionales.

## MÉTODO

Se trata de un **ensayo teórico-reflexivo** estructurado para responder a la pregunta: ¿*la* USG *realizada por enfermeros, en el* ámbito *del PE, debe entenderse como un componente de la etapa de evaluación o como una intervención de enfermería?*

### Referencial Teórico-Metodológico

La reflexión se basó en el modelo de organización del PE (ADPIE) como estructura lógico-procedimental de la atención; en los SLP en enfermería (NANDA-I, NIC, NOC), como ontologías operativas que vinculan diagnóstico-resultado-intervención; en la literatura de representación/modelización científica, para discutir los límites y la precisión representacional de los modelos cuando se confrontan con la práctica; y aportaciones sobre el razonamiento clínico en enfermería (analítico frente a no analítico/por patrones), para explicitar cómo las decisiones a pie de cama pueden tensar las fronteras conceptuales entre evaluar e intervenir.

### Corpus Teórico

El *corpus* reunió: (a) normativas profesionales pertinentes a la USG por parte de enfermeros y al desarrollo obligatorio del PE; (b) ediciones vigentes de las clasificaciones NANDA-I, NIC y NOC; (c) publicaciones sobre USG en la atención de enfermería/POCUS y sobre el desarrollo/validación de intervenciones de monitorización; (d) obras de referencia en representación científica y razonamiento clínico. La suficiencia del corpus se guió por la saturación teórica referida a la pregunta orientadora.

### Estrategia Analítica

El análisis combinó: (1) análisis conceptual (aclaración de términos y relaciones entre categorías NANDA-I/NIC/NOC en el contexto del ADPIE); (2) contraste de escenarios (USG como extensión de la evaluación frente a USG como intervención de enfermería, con enfoque en intervenciones de monitorización); (3) un experimento mental con caso clínico hipotético en terapia intensiva, utilizado como “dispositivo de prueba” para evaluar el rendimiento explicativo del modelo ADPIE en cada marco; y (4) análisis de consecuencias (implicaciones de cada marco para la enseñanza, la práctica clínica y posibles ajustes en las clasificaciones).

### Construcción y Uso del Caso Hipotético

El caso se construyó a partir de situaciones frecuentes en la práctica hospitalaria (postoperatorio, riesgo de retención urinaria, decisión sobre cateterismo vesical). Se explicaron los datos clínicos, los diagnósticos de enfermería elegibles, los resultados esperados y las posibles intervenciones. La USG se incorporó como paso decisivo para demostrar, de manera comparativa, cómo el marco, la **avaluación** o la **intervención** alteran el flujo lógico del PE, la valoración de los indicadores de la NOC y la selección de intervenciones en la NIC.

### Criterios de Rigor y Credibilidad

Para garantizar la calidad teórica, se adoptaron: coherencia interna del argumento; transparencia en la cadena de inferencias; audibilidad de las fuentes (citas directas a clasificaciones y normativas); triangulación conceptual entre ADPIE, NANDA-I/NIC/NOC y literatura de modelización científica; y búsqueda deliberada de contraejemplos (por ejemplo, situaciones en las que la USG produce un “efecto indirecto” sobre los resultados).

### Aspectos Éticos

Al tratarse de un ensayo de carácter teórico-reflexivo, esta investigación no se sometió a la apreciación de un comité de ética. El caso clínico utilizado para la ilustración es hipotético y no se refiere a ningún paciente real. Además, se respetan rigurosamente las buenas prácticas académicas para la producción de conocimiento, asegurando la cita correcta y la referencia adecuada de todas las fuentes utilizadas.

## ¿LA ULTRASONOGRAFÍA FORMA PARTE DEL EXAMEN FÍSICO DE ENFERMERÍA, COMO PARTE DE LA EVALUACIÓN DEL PACIENTE EN LA CAMA?

La USG permite visualizar e interpretar las estructuras del cuerpo humano, caracterizándolas y permitiendo inferencias sobre su funcionamiento. Esto, per se, se caracteriza como una extensión del examen físico^(6)^. La avaluación de enfermería puede realizarse con el uso de instrumentos, incluidos los exámenes de imagen^(2)^. La NANDA-I describe de forma sistemática y estructurada todo el proceso de razonamiento desde la evaluación hasta la identificación del DE prioritario, pudiendo incluir la USG en la avaluación de detección y la avaluación en profundidad^(3)^.

La avaluación de detección se considera la fase inicial de la avaluación, en la que se recogen los primeros datos y se inicia el razonamiento clínico. Por lo tanto, la USG puede enriquecer este momento como un artefacto que apoya y califica el razonamiento clínico^(3)^. A partir de los datos de detección se orienta y profundiza el proceso de avaluación, lo que da como resultado un DE^(3)^. Si la USG se realiza en respuesta a una necesidad percibida en la detección, esta puede aportar información objetiva que oriente hacia DE específicos. Esto también permite percibir que la NANDA-I preconiza el pensamiento hipotético-deductivo, al prever el uso de procedimientos específicos de recolección de datos para confirmar o refutar hipótesis diagnósticas^(3)^.

Además de los argumentos ya expuestos, es importante comprender cómo se inserta la USG en el modelo ADPIE en esta línea argumentativa. En un caso hipotético, un enfermero llega a la UTI a las 13:00 horas para un turno de seis horas. Se le asigna el caso de un paciente varón de 80 años que ha sido sometido a una cirugía abdominal, está postrado en cama y presenta como comorbilidad hiperplasia prostática benigna (HPB). Al avaluar al paciente, el enfermero se da cuenta de que se le ha retirado el catéter vesical de demora (CVD) y encuentra en los registros de enfermería que esto ocurrió a las 12:00 horas (letra A - Avaliação en portugués - del modelo ADPIE). Se definió el DE “Riesgo de retención urinaria (00322)”, cuyos factores de riesgo en este caso son “postura inadecuada en el inodoro” y “privacidad inadecuada”, y como condición asociada, “enfermedades prostáticas”.

Esto conforma la etapa de diagnóstico, letra D^(3)^. En la etapa de planeamiento (letra P), el enfermero enumera el resultado de la NOC “Eliminación urinaria (0503)” y la intervención “Asistencia en el autocuidado: uso del inodoro (1804)” de la NIC^(4,5)^. Esta intervención incluye ayuda en la eliminación urinaria, suministro de dispositivos auxiliares cuando sea necesario y garantía de privacidad. Los indicadores clínicos valorados en una escala Likert de 5 puntos, en la que cuanto mayor es la puntuación, más deseable es el estado, se describen en la Cuadro 1 ^(5)^.

**Cuadro 1:** Indicadores del resultado de enfermería “Eliminación urinaria (0503)” elegidos para el caso hipotético – Florianópolis, SC, Brasil, 2025.

Indicador	Escala de medición				
	1. Gravemente comprometido	2. Muy comprometido	3. Moderadamente comprometido	4. Levemente comprometido	5. No comprometido
Reconocimiento de deseo de orinar					X O
Patrón de eliminación					X O
Cantidad de orina					X O
Vacía completamente la vejiga					X O
	1 Grave	2 Sustancial	3 Moderado	4 Leve	5 Ninguno
Retención urinaria					X O

Nota: “X” representa el estado actual del paciente y “O”, el estado esperado. Fuente: Adaptado de Moorhead, Johnson y Swanson (2024, p. 408–409)^(5)^.

La implementación del plan de cuidados durante el turno de enfermería en este caso hipotético se considera concluida, ya que las actividades de cuidado se desarrollaron sin particularidades dignas de mención. Así, se compone la etapa I.

La evolución de la enfermería, etapa siguiente y final del PE, se describe como la “evaluación de los resultados alcanzados en materia de enfermería y salud de la persona, la familia, la colectividad y los grupos especiales”^(2)^, y considera que esta etapa “permite el análisis y la revisión de todo el proceso de enfermería”^(2)^. Es en la etapa de evolución donde se valoran los indicadores de los resultados de enfermería y se analiza el comportamiento de estos indicadores a partir del plan de cuidados que se ha ejecutado^(2,5)^ (Cuadro 2).

**Cuadro 2:** Puntuaciones obtenidas en los indicadores de resultados de enfermería “Eliminación urinaria (0503)” evaluados a las 18 horas – Florianópolis, SC, Brasil, 2025.

Indicador	Escala de medición					
	1. Gravemente comprometido	2. Muy comprometido	3. Moderadamente comprometido	4. Levemente comprometido	5. No comprometido	NA*
Reconocimiento de deseo de orinar	X					
Patrón de eliminación	X					
Cantidad de orina						X
Vacía completamente la vejiga	X					
	1 Grave	2 Sustancial	3 Moderado	4 Leve	5 Ninguno	
Retención urinaria	X					

Nota: NA*: No evaluado. “X” representa la puntuación obtenida. Fuente: Adaptado de Moorhead, Johnson y Swanson (2024, p. 408–409)^(5)^.

En la evaluación de las 18:00 horas, el paciente presentó una puntuación de 1 en “reconocimiento de deseo de orinar” por la ausencia de deseo miccional y “patrón de eliminación”, ya que no presentó diuresis en seis horas^(5)^. No se evaluó la “cantidad de orina”, ya que el paciente no orinó, y la evaluación de la región suprapúbica mediante palpación y percusión se vio dificultada por la herida quirúrgica abdominal. El hecho de que el paciente no vaciara la vejiga y, al ofrecerle un orinal, dijera que no podía orinar, justifica la puntuación 1 en los indicadores “vaciar la vejiga completamente” y “retención urinaria”^(5)^.

El enfermero decide entonces evaluar al paciente con una USG. Cuando esto ocurre, se reinicia lo PE, retomando la primera etapa del ADPIE. Al realizar la USG de la vejiga, se constata que hay una cantidad importante de orina retenida en la vejiga. Esto permite identificar la forma en que la USG se relaciona con la etapa de “avaluación en profundidad” de la NANDA-I, al subsidiar el razonamiento clínico y el diagnóstico^(3)^.

Así, el DE cambia a “Eliminación urinaria alterada (00016)”, que se define como “incapacidad para excretar eficazmente los fluidos y residuos almacenados en la vejiga a través de la uretra”^(3)^. La característica definitoria encontrada es “retención urinaria”, teniendo como factores relacionados “postura inadecuada en el inodoro” e “inadecuada privacidad”^(3)^. El paciente se encuentra además en una población de riesgo, por ser anciano, y tiene como condición asociada la HPB^(3)^.

En la etapa de planificación, el enfermero revisa el cuadro clínico para identificar el estado actual del paciente y enumerar las intervenciones que permitan alcanzar los resultados esperados^(2)^. El estado actual del paciente es el mismo que se presenta en la Cuadro 2, al que se añaden los hallazgos obtenidos mediante la USG (Cuadro 3).

**Cuadro 3:** Etapa de planificación tras la evaluación con USG de la vejiga – Florianópolis, SC, Brasil, 2025.

Indicador	Escala de medición				
	1. Gravemente comprometido	2. Muy comprometido	3. Moderadamente comprometido	4. Levemente comprometido	5. No comprometido
Reconocimiento de deseo de orinar	X				O
Patrón de eliminación	X				O
Cantidad de orina					X O
Vacía completamente la vejiga	X				O
	1 Grave	2 Sustancial	3 Moderado	4 Leve	5 Ninguno
Retención urinaria	X				O

Nota: “X” representa el estado actual del paciente y “O”, el estado esperado. Fuente: Adaptado de Moorhead, Johnson y Swanson (2024, p. 408–409)^(5)^.

Se produjo un cambio en los indicadores evaluados tras la aplicación de la USG en el caso. El indicador “cantidad de orina”, que no se había evaluado, pasó a tener una puntuación de 5, ya que la USG permitió medir el volumen vesical de forma no invasiva. Además, permitió evaluar de forma más consistente los indicadores “vaciado completo de la vejiga” y “retención urinaria”^(5)^.

De este modo, el enfermero decide realizar la intervención “Cateterismo vesical (0580)”, dirigiendo la intervención a la característica definitoria “retención urinaria” y ya no a los factores relacionados^(3,4)^. El volumen de orina drenado fue de 550 mililitros. A partir de ahí, es posible reevaluar las puntuaciones obtenidas en cada indicador clínico del resultado “Eliminación urinaria (0503)”^5^ (Cuadro 4).

**Cuadro 4:** Puntuaciones obtenidas en los indicadores clínicos evaluados en el caso tras la realización de un cateterismo vesical – Florianópolis, SC, Brasil, 2025.

Indicador	Escala de medición				
	1. Gravemente comprometido	2. Muy comprometido	3. Moderadamente comprometido	4. Levemente comprometido	5. No comprometido
Reconocimiento de deseo de orinar	X				
Patrón de eliminación			X		
Cantidad de orina					X
Vacía completamente la vejiga					X
	1 Grave	2 Sustancial	3 Moderado	4 Leve	5 Ninguno
Retención urinaria					X

Nota: “X” representa o escore alcançado. Fonte: Adaptado de Moorhead, Johnson e Swanson (2024, p. 408–409)^(5)^.

En este punto, nuevamente en la etapa E del modelo ADPIE, se comparan los resultados obtenidos. El indicador “reconocimiento de la necesidad” sigue siendo 1, ya que el paciente presentaba retención de orina en la vejiga y no refería necesidad de orinar ni molestias. El “patrón de eliminación” se restablece parcialmente a partir de la cateterización, evaluado como moderadamente comprometido (puntuación 3). El indicador “cantidad de orina” no se modificó, ya que la USG mostró una cantidad adecuada de orina, lo que se confirmó con la cateterización. Por su parte, los indicadores “vacía completamente la vejiga” y “retención urinaria” se resuelven por completo a partir de la intervención realizada. Esto concluye la descripción del ejercicio mental con aplicación de la USG en el modelo ADPIE en la primera etapa del PE, la evaluación^(5)^.

El caso demuestra que la USG encaja en la etapa de evaluación. Además, la USG no cumple con la definición de intervención de enfermería de la NIC, que es “cualquier tratamiento que, basado en el juicio y el conocimiento clínico, un enfermero pone en práctica para mejorar los resultados del paciente”^(4)^. En este sentido, la USG no es un “tratamiento”, ya que no modificó directamente los resultados de enfermería.

## ¿LA ULTRASONOGRAFÍA ES UNA INTERVENCIÓN DE ENFERMERÍA?

Desde 1996, la NIC considera que la USG realizada por enfermeros es una intervención de enfermería: “Ultrasonografía: obstétrica y ginecológica (6982)”^(4)^. Actualmente, en la octava edición, hay una segunda intervención de enfermería con uso de USG: “Ultrasonografía: vejiga (0565)”^(4,5,6,7)^. Esta última se ha desarrollado recientemente^(7)^ y se define como “realización de exámenes por ecografía para determinar la función o la estructura de la vejiga”^(4)^.

Además de la definición de la NIC para la intervención de enfermería, la NANDA-I, al describir el Modelo Tripartito de Kamitsuru, señala que “las acciones o intervenciones autónomas de enfermería son *tratamientos* basados en patrones de conocimiento de enfermería, determinados por el enfermero como adecuados para abordar los factores etiológicos de un DE o manejar los síntomas”^(3)^ (énfasis nuestro). En la presente línea argumentativa, la USG puede considerarse un tratamiento, ya que se dirige al DE y mejora los resultados.

El término “tratamiento” no se describe formalmente en la NIC ni en la clasificación de la NANDA-I. En el Diccionario Online de Portugués, uno de los significados de la entrada “tratamiento” es “conjunto de medios prácticos para combatir una enfermedad; terapéutica”^(8)^. Por su parte, el Diccionario Michaelis, también en línea, ofrece como uno de los significados de esta misma entrada “conjunto de medios que utiliza el médico para curar o aliviar al enfermo”^(9)^.

En común, los significados lingüísticos del término “tratamiento” incluyen el “conjunto de medios”. En este sentido, la USG realizada por enfermeros constituye el “conjunto de medios” y es, en sí misma, también un “conjunto de medios” o acciones que permiten obtener mejores resultados, siendo este su “efecto terapéutico”, que denota la idea de “tratamiento”. Es importante destacar también que este “efecto”, en este caso, no es directo, sino indirecto. Esto se debe a que las intervenciones con el uso de la USG pueden considerarse intervenciones de monitorización, tal y como se describe en la NIC^(4)^.

La NIC incluye numerosas intervenciones de monitoreo, como el monitoreo ácido-básico y de los signos vitales, que incorporan y reflejan el juicio clínico e indican al enfermero qué debe buscar y qué hacer cuando se produce algún evento^(4)^.

Además, es importante destacar que los diagnósticos de riesgo requieren intervenciones de monitoreo. La NANDA-I define los DE de riesgo como la susceptibilidad de desarrollar, en el futuro, una respuesta humana indeseable^(3)^. Además de actuar para controlar los factores de riesgo, también es necesario identificar la ocurrencia del evento en riesgo. En un ejemplo rápido, el diagnóstico “Riesgo de lesión por presión en adultos (00304)” puede modificarse o atenuarse directamente mediante la intervención “Prevención de lesiones por presión (3540)” a partir del control de los factores de riesgo, y la ocurrencia del diagnóstico centrado en el problema — “Lesión por presión en adultos (00312)” — puede verificarse mediante la intervención de monitoreo “Supervisión de la piel (3590)”^(3,4)^.

A modo de ejemplo, para llevar a cabo la intervención “Monitorización de signos vitales (6680)”, es necesario dominar técnicas como la medición de la presión arterial, la saturación periférica de oxígeno, la temperatura corporal, la frecuencia cardíaca, etc^(4)^. Este “saber hacer” es lo que delimita la intervención. Se trata de una intervención precedida por un razonamiento clínico que la fundamenta y la provoca en el plano asistencial para obtener resultados específicos^(4)^.

A diferencia de la sección anterior, se realizará el ejercicio mental de encajar la USG en el modelo ADPIE, esta vez como intervención de enfermería, utilizando el mismo caso hipotético. Así, con el DE “Riesgo de retención urinaria (00322)”, NOC “Eliminación urinaria (0503)” y NIC “Asistencia en el autocuidado: uso del inodoro (1804)”^(3,4,5)^, a las 18 horas del día en cuestión, el paciente presenta las puntuaciones evaluadas ya descritas en la Cuadro 2.

Al evaluar estas puntuaciones, el enfermero constata que el DE del paciente sigue siendo el mismo, “Riesgo de retención urinaria (00322)”^(3)^. Y esto se debe a que se puede considerar que no hay indicios suficientes que justifiquen su cambio, ya que no han surgido nuevos factores de riesgo y los ya existentes no se han resuelto. Además, la ocurrencia de retención urinaria puede evaluarse mejor con el uso de la intervención de enfermería “Ultrasonografía: vejiga (0565)”, ya que, así como es posible que el paciente presente “Eliminación urinaria alterada (00016)”, también es posible que haya habido una reducción en la producción de orina^(3,4)^. La diferencia es que, en un caso, la realización de una cateterización vesical es una intervención que puede considerarse; en el otro, no.

De este modo, para alcanzar el resultado “Eliminación urinaria (0503)”, el enfermero incluye la intervención de enfermería “Ultrasonografía: vejiga (0565)”. En esta etapa de planificación, también se podría incluir la intervención “Cateterismo vesical (0580)”, que se realizaría en función del resultado obtenido por la intervención “Ultrasonografía: vejiga (0565)”^(4,5)^. Por lo tanto, en este ejercicio, en este momento, se propone realizar únicamente la intervención “Ultrasonografía: vejiga (0565)”^(4)^ (Cuadro 5).

**Cuadro 5:** Estado actual del paciente antes de la realización de la intervención de enfermería “Ultrasonografía: vejiga (0565)” y estado esperado – Florianópolis, SC, Brasil, 2025.

Indicador	Escala de medición					
	1. Gravemente comprometido	2. Muy comprometido	3. Moderadamente comprometido	4. Levemente comprometido	5. No comprometido	NA*
Reconocimiento de deseo de orinar	X				O	
Patrón de eliminación	X				O	
Cantidad de orina					O	X
Vacía completamente la vejiga	X				O	
	1 Grave	2 Substancial	3 Moderado	4 Leve	5 Nenhum	NA*
Retención urinaria	X				O	

Nota: NA*: No evaluado. “X” representa el estado actual del paciente y “O”, el estado esperado. Fuente: Adaptado de Moorhead, Johnson y Swanson (2024, p. 408–409)^(5)^.

A diferencia de la línea argumentativa anterior, se observa que el estado actual es el mismo que el de la etapa de evolución del ciclo que inició el presente ejercicio. La etapa de implementación se lleva a cabo mediante la intervención “Ultrasonografía: vejiga (0565)”^(4)^. Al realizar la intervención, el enfermero identificó un aumento del volumen urinario a partir de la USG. Así, el resultado de enfermería puede volver a valorarse (Cuadro 6).

**Cuadro 6:** Puntuaciones obtenidas por el paciente tras la realización de la intervención de enfermería “Ultrasonografía: vejiga (0565)” – Florianópolis, SC, Brasil, 2025.

Indicador	Escala de medición					
	1. Gravemente comprometido	2. Muy comprometido	3. Moderadamente comprometido	4. Levemente comprometido	5. No comprometido	NA*
Reconocimiento de deseo de orinar	X					
Patrón de eliminación	X					
Cantidad de orina					X	
Vacía completamente la vejiga	X					
	1 Grave	2 Sustancial	3 Moderado	4 Leve	5 Ninguno	NA*
Retención urinaria	X					

Nota: NA*: No evaluado. “X” representa la puntuación obtenida. Fuente: Adaptado de Moorhead, Johnson y Swanson (2024, p. 408–409)^(5)^.

A partir de las puntuaciones obtenidas, se puede identificar que el indicador “cantidad de orina” tras la realización de la USG puede evaluarse y es adecuado. Los indicadores “vaciado completo de la vejiga” y “retención urinaria” pueden evaluarse de forma más consistente, lo que permite una mejor toma de decisiones^(5)^.

Al igual que en el caso presentado en la sección anterior, el DE caracterizado es entonces “Eliminación urinaria alterada (00016)”, lo que culmina en el mismo resultado de enfermería “Eliminación urinaria (0503)” y la intervención “Cateterismo vesical (0580)”^(3,4,5)^. Una vez realizada la cateterización, los indicadores evaluados en la etapa de evolución muestran la misma mejora que se presenta en la Cuadro 4.

Es importante reforzar que la USG como intervención modifica los resultados obtenidos. En el caso hipotético, la mejora de los resultados se produjo de forma indirecta, al modificar el plan de cuidados e identificar la necesidad de la cateterización vesical^(4)^.

En este caso, suponiendo que la USG demostrara que la vejiga del paciente estaba vacía, el indicador “cantidad de orina” se mantendría en la puntuación 1; el indicador “vacía la vejiga completamente” no se evaluaría, ya que el paciente no tiene contenido intravesical que vaciar; el indicador “retención urinaria”, que tenía una puntuación de 1, se puntuaría con 5, ya que no habría orina retenida en la vejiga. De este modo, no se realizaría la cateterización vesical^(5)^.

En este sentido, en el caso de la USG vesical, existe un beneficio importante en la constatación de “no ocurrencia” o en la “prueba negativa”, evitando que el paciente se someta a un cateterismo vesical innecesario. Los estudios demuestran que el uso de la USG por parte del personal de enfermería permite indicar la necesidad de un cateterismo vesical, lo que reduce la incidencia de las posibles complicaciones de este procedimiento, especialmente la infección del tracto urinario^(10,11)^.

## DISCUSIÓN

Las aplicaciones de la USG en la práctica de la enfermería son diversas y pueden interpretarse en ambas etapas del PE. Además, este “doble encaje” no es algo inédito, ya que también ocurre con otras acciones de enfermería^(4)^. La realidad es más compleja que las cinco etapas que involucran el modelo ADPIE, la práctica de enfermería es dinámica y los procesos de razonamiento, toma de decisiones y acción ocurren de forma simultánea y con diversos recursos y recorridos de pensamiento empleados.

Un modelo es una construcción teórica o real que representa aspectos del mundo real o permite razonar sobre ese mundo^(12)^. Pero, ¿qué hace que el modelo ADPIE represente el PE? Todo modelo es una representación idealizada de un objetivo, que es lo que se pretende representar. Al ser idealizada, esta representación no necesita ser absoluta, es decir, el modelo solo tiene que corresponder al objetivo en algunos aspectos clave^(12)^.

En este sentido, aunque el modelo ADPIE no representa el PE de forma exacta, permite obtener y producir conocimientos. En el entorno real de la atención, el PE no existe como etapas estructuradas, secuenciales, interdependientes, ordenadas, cíclicas, graduales y lineales, pero puede explicarse de esa manera. En el escenario asistencial, los enfermeros evalúan al paciente, al mismo tiempo que intervienen y evalúan los resultados, generando simultáneamente nuevos diagnósticos, de forma dinámica, rápida y objetiva^(12,13,14)^.

La NANDA-I describe que, a partir de la repetición y la familiaridad con el contexto y las situaciones clínicas vividas, un enfermero experimentado puede pasar rápidamente por una etapa del PE^(3)^. Esto se debe a que los enfermeros experimentados tienden a utilizar el razonamiento no analítico, por reconocimiento de patrones, lo que conduce a una toma de decisiones más rápida^(15)^. La práctica de la enfermería exige un pensamiento relacional para comprender la compleja maraña de información que rodea los problemas del paciente ^(4,13)^.

En el caso hipotético mencionado anteriormente, esta posible limitación representacional del modelo ADPIE aparece cuando indica que la USG es una intervención de monitoreo. Esto se debe a que, al realizar una intervención de monitoreo, cuando se produce un evento que representa un cambio en el DE, las etapas posteriores (evolución y avaluación) pueden no ser necesarias, dando la impresión de que no se desarrollarán.

Cuando se realiza la USG de la vejiga y se constata el elevado volumen urinario retenido en la vejiga, se comprueba la ocurrencia del indicador “retención urinaria”, que es una característica definitoria del DE “Eliminación urinaria alterada (00016)”^(3)^. Es decir, se salta de la etapa de implementación directamente a la etapa de diagnóstico. Esto representa el razonamiento no analítico que, al reconocer un patrón, estructura automáticamente las conexiones entre los acontecimientos en la realidad basándose en la experiencia previa^(15)^.

Esta posible limitación del modelo ADPIE se supera tras una breve reflexión. Cuando hay un hallazgo facilitado por la USG, se identifica y valora automáticamente el resultado de enfermería, lo que permite llegar al diagnóstico centrado en el problema. Por más rápido que pueda parecer, las etapas se producen. De este modo, se puede afirmar que el problema de representatividad del modelo ADPIE no es necesariamente ese.

El principal problema con el modelo ADPIE y la USG que se presenta aquí es la ambigüedad representacional, un problema de precisión del modelo. Esta es una condición explorada en el modelado científico y se produce porque, como ya se ha dicho, los modelos no son representaciones absolutas de sus objetivos, lo que puede dar lugar a representaciones inexactas en algunos aspectos^(12)^. Esto no inutiliza un modelo, solo presenta una limitación en una situación determinada analizada^(12)^.

Lo que ocurre en el caso aquí descrito es que la realidad representada es la misma en ambos argumentos, el mismo paciente hipotético, la misma situación clínica, pero con dos representaciones posibles por el modelo ADPIE. En los ejercicios mentales desarrollados, ya sea considerando la USG como componente de la etapa A o de las etapas P e I, el modelo funciona adecuadamente.

Una realidad, dos representaciones posibles. Además, en el caso de que la USG se considere una intervención de enfermería, es necesario repetir el proceso más veces, lo que puede corregirse dejando de asumir la hipótesis inicial de analizar una situación clínica de forma aislada e incorporando otros diagnósticos, intervenciones y resultados en el plan de cuidados.

Además, al considerar la USG como componente de la etapa de avaluación o como una intervención, en ambos casos existen posibles ramificaciones que deben discutirse. Asumir un lado u otro de esta discusión exige acciones o desencadena una cascada de preguntas que deben responderse para mantener la solidez teórica del PE con el uso de SLP.

En el primer caso, asumir que la USG no es una intervención puede significar asumir que otras intervenciones de monitorización descritas en la NIC tampoco son intervenciones. Estas intervenciones no modifican los factores relacionados o de riesgo de los DE, pero subsidian y aumentan la precisión diagnóstica, lo que permite que estas acciones sean cuestionadas por no tener un efecto terapéutico directo. Esto podría desencadenar una revisión amplia y compleja de toda la NIC.

En el segundo caso, al asumir que la USG es una intervención de enfermería, se asume que se trata de un “tratamiento”, tal y como se describe en la definición de intervención de la NIC^(4)^. En este sentido, puede ser necesario definir mejor en la NIC el término “tratamiento” o incluso desarrollar una categorización de las intervenciones de enfermería según su objetivo, contemplando las de monitorización. También es posible plantear un cambio en la definición general de intervención de enfermería de la NIC, lo que permitiría contemplar las intervenciones de monitorización.

Aun en este caso, existe una posible complicación problemática que puede ocurrir al asumir que la USG es una intervención, denominada aquí “intervencionismo”. Esto significa que es posible que otras acciones que se utilizan como pruebas clínicas también se consideren intervenciones, como es el caso de la auscultación, la percusión y la palpación. Del mismo modo que la USG puede estar precedida y respaldada por el razonamiento clínico, la auscultación pulmonar, por ejemplo, también puede ocurrir de la misma manera.

Además, la reflexión presentada también apunta a un posible impacto en la enseñanza de la enfermería que merece atención. El aprendizaje de la USG por parte de los enfermeros aún se encuentra en una fase incipiente en Brasil. El COFEN, por ejemplo, determina la necesidad de formación, pero no especifica cómo debe llevarse a cabo este proceso formativo^(1)^.

Por lo tanto, se puede conjeturar que, al considerar la USG como parte integrante del examen físico de enfermería, es decir, como una herramienta semiológica utilizada en la etapa de avaluación, esta puede situarse fuera del ámbito exclusivo de las especialidades. Este enfoque sugiere su inclusión ya en la formación inicial, a través de disciplinas como “fundamentos de enfermería”, aún en la licenciatura. Por otro lado, al interpretarse como una intervención, la USG puede entenderse desde una perspectiva especializada, lo que refuerza la necesidad de una capacitación específica y cualificada, considerando incluso su naturaleza dependiente del operador y exigiendo una curva de aprendizaje adecuada.

El encuadre de la USG en el PE a partir del modelo ADPIE revela no solo la complejidad de la práctica de enfermería, sino también la necesidad de un análisis crítico de los modelos teóricos que la orientan. El modelo ADPIE, a pesar de ser útil para organizar y racionalizar el PE, presenta limitaciones al intentar representar la dinámica del cuidado en su totalidad. La ambigüedad representacional expone las limitaciones del modelo para captar la fluidez y la simultaneidad de las acciones de enfermería.

Por lo tanto, lejos de cerrar la discusión, este ensayo busca ampliar el debate sobre cómo las prácticas innovadoras, como la USG realizada por enfermeros, se articulan conceptualmente en el ámbito del PE. Más que definir si se trata de una intervención o de una herramienta de avaluación, la propuesta aquí es reconocer que la práctica de la enfermería desafía los modelos teóricos, al presentar situaciones complejas que exigen interpretaciones flexibles y críticas. Esta tensión entre la práctica y la teoría no representa una debilidad: es precisamente a través de este enfrentamiento que la ciencia de la enfermería se renueva, amplía sus límites y afirma su relevancia frente a las transformaciones contemporáneas de la atención.

## CONSIDERACIONES FINALES

Este ensayo ha demostrado que la incorporación de la USG en la práctica de la enfermería desafía los paradigmas teóricos del PE, revelando una dualidad importante: la USG puede actuar tanto como herramienta de avaluación como de intervención. Esta ambigüedad expone las limitaciones del modelo ADPIE para captar la dinámica no lineal y relacional de la atención clínica real, especialmente en situaciones que requieren un razonamiento rápido.

Como implicaciones, este estudio apunta a posibles revisiones en los sistemas de clasificación de enfermería, particularmente en la NIC, para acomodar mejor las intervenciones de monitoreo. Paralelamente, aunque el ADPIE mantiene su utilidad como estructura teórica, su aplicación requiere flexibilidad para enfoques no lineales de toma de decisiones. Para avanzar en esta discusión, futuras investigaciones pueden investigar empíricamente el comportamiento de la USG en el PE.

## Data Availability

Todo el conjunto de datos que apoya los resultados de este estudio fue publicado en el propio artículo.
